# Counterintuitive Electrostatics upon Metal Ion Coordination to a Receptor with Two Homotopic Binding Sites

**DOI:** 10.1021/jacs.1c08507

**Published:** 2022-02-10

**Authors:** Vidar Aspelin, Anna Lidskog, Carlos Solano Arribas, Stefan Hervø-Hansen, Björn Stenqvist, Richard Chudoba, Kenneth Wärnmark, Mikael Lund

**Affiliations:** † Division of Theoretical Chemistry, Department of Chemistry, Lund University, Lund SE 221 00, Sweden; ‡ Center for Analysis and Synthesis (CAS), Department of Chemistry, Lund University, Lund SE 221 00, Sweden; ¶ Division of Physical Chemistry, Department of Chemistry, Lund University, Lund SE 221 00, Sweden; § Lund Institute of Advanced Neutron and X-ray Science (LINXS), Scheelevägen 19, Lund SE 223 70, Sweden

## Abstract

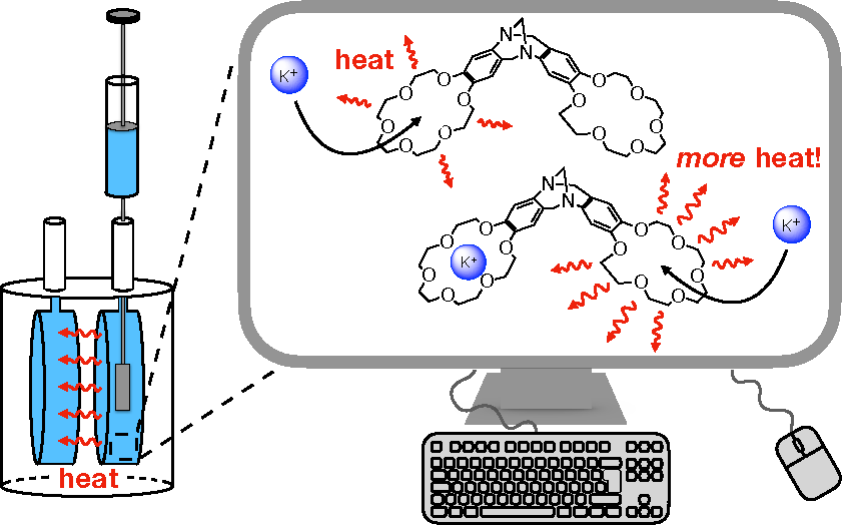

The consecutive binding of two potassium ions to a bis(18-crown-6) analogue of Tröger’s base (BCETB) in water was studied by isothermal titration calorimetry using four different salts, KCl, KI, KSCN, and K_2_SO_4_. A counterintuitive result was observed: the enthalpy change associated with the binding of the second ion is more negative than that of the first (Δ*H*_bind,2_^°^ < Δ*H*_bind,1_^°^). This remarkable finding is supported by continuum electrostatic theory as well as by atomic scale replica exchange molecular dynamics simulations, where the latter robustly reproduces experimental trends for all simulated salts, KCl, KI, and KSCN, using multiple force fields. While an enthalpic K^+^–K^+^*attraction* in water poses a small, but fundamentally important, contribution to the overall interaction, the probability of the collapsed conformation (COL) of BCETB, where both crown ether moieties (CEs) of BCETB are bent in toward the cavity, was found to increase successively upon binding of the first and second potassium ions. The promotion of the COL conformation reveals favorable intrinsic interactions between the potassium coordinated CEs, which further contribute to the observation that Δ*H*_bind,2_^°^ < Δ*H*_bind,1_^°^. While the observed trend is independent of the counterion, the origin of the significantly larger magnitude of the difference Δ*H*_bind,2_^°^ – Δ*H*_bind,1_^°^ observed experimentally for KSCN was studied in light of the weaker hydration of the thiocyanate anion, resulting in an enrichment of thiocyanate ions close to BCETB compared to the other studied counterions.

## Introduction

Receptors are proteins located inside or on the surface of cells that can receive and transduce chemical signals. Upon binding of an external ligand, a conformational change is triggered in the receptor, which in turn activates a physiological function.^1^ Due to the abundance and importance of receptors in biological systems, the development and application of synthetic receptors has received much attention. One objective for the development of synthetic receptors is to provide means to systematically study the fundamental thermodynamic factors governing receptor/ligand associations.^2^ This includes the investigation of concepts such as entropy–enthalpy compensation^3,4^ and binding cooperativity,^5^ concepts of fundamental importance in biological receptors.

One recognition motif commonly utilized in synthetic receptors is the crown ether (CE). Crown ethers are macrocyclic oligomers of ethylene oxide capable of selectively binding cations.^6^ By altering the size of the cavity, CEs can be tuned to recognize ions of a certain size, and, as a result, they have found applications in many areas within host–guest chemistry, including the design of ion-selective electrodes,^7−9^ recovery of cesium from nuclear waste,^10,11^ and drug delivery.^12−14^

**Figure 1:**
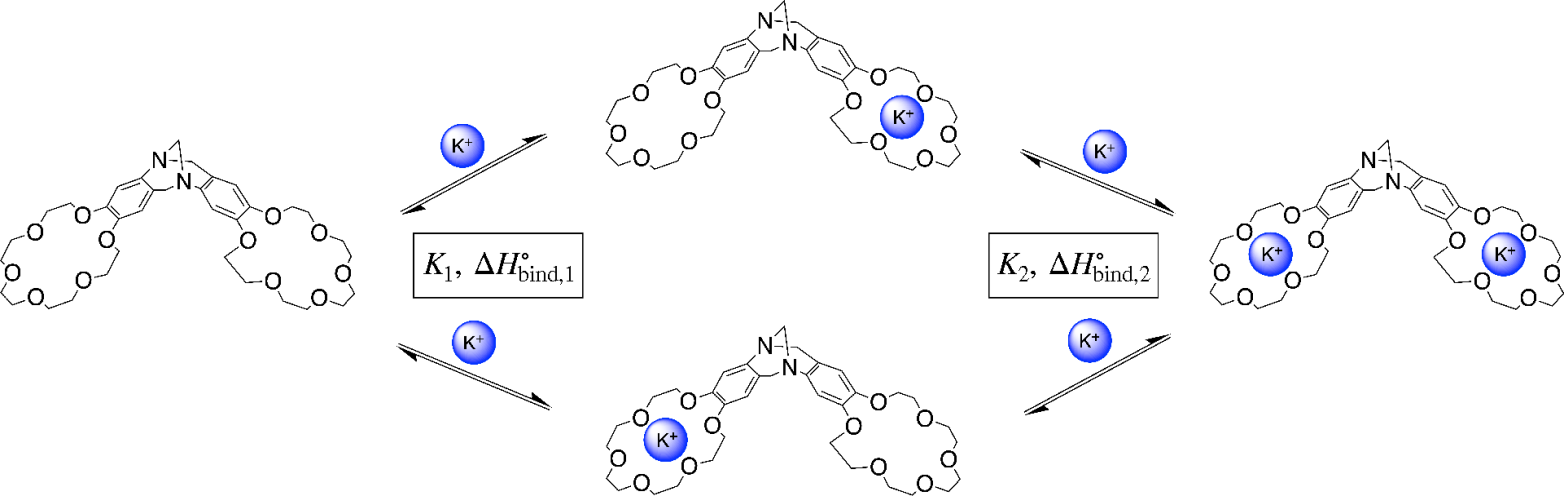
Sequential binding of potassium ions to a bis(18-crown-6) analogue of Tröger’s base (BCETB).

While the majority of the studies of CE-based synthetic receptors concern receptors containing a single CE, we herein present a study of the thermodynamics governing the binding of potassium ions to a ditopic bis(18-crown-6) analogue of Tröger’s base (BCETB, Figure 1).^15,16^ The association of multiple cations to a single, multitopic receptor represents an interesting thermodynamic system, with many different factors contributing to the overall stability of the complex. Previous studies of host–guest complexes containing two metal cations have employed interaction models including cation–cation repulsion and solvation effects to explain experimental observations.^17−19^ Overall, considering also the interactions within the complex, the absolute binding affinity of the cation relies on a balance between the desolvation of the ion and the binding site and the subsequent formation of intermolecular interactions when the ion is fixed in the binding site, involving both interactions within the complex and interactions between the complex and the solvent.

We have here estimated the standard free energies and enthalpies for the consecutive binding of two potassium ions to BCETB using isothermal titration calorimetry (ITC).^20^ The ITC experiments were performed using KCl, KI, KSCN, and K_2_SO_4_ in order to account for the possible influence of the counterion on the binding thermodynamics. To elucidate the origin of the observed difference in the binding enthalpy of the first and second potassium ion, we have employed continuum electrostatic theory and replica exchange molecular dynamics (REMD) simulations.^21^

## Results and Discussion

### Isothermal Titration Calorimetry

**Figure 2:**
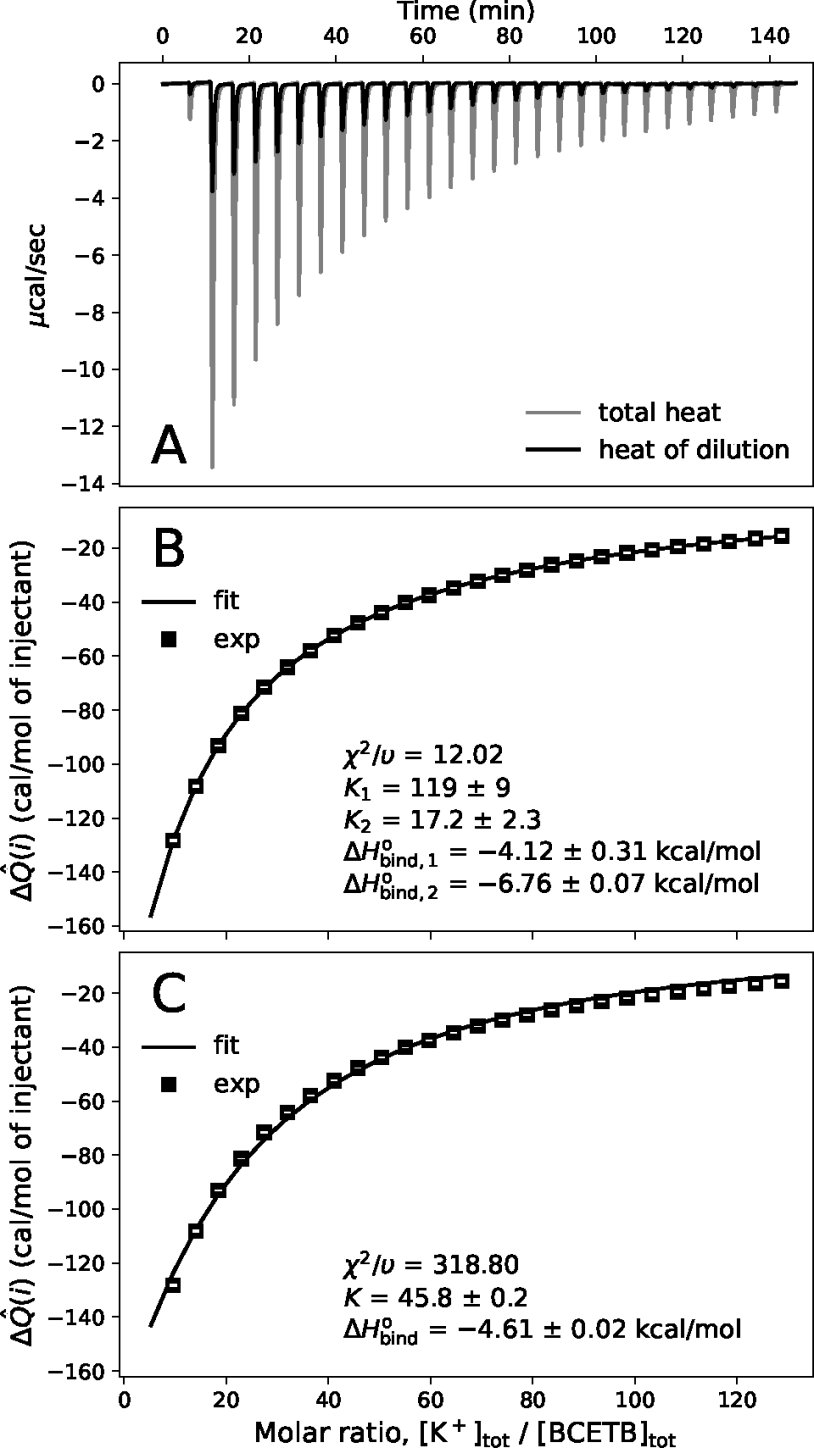
Normalized, integrated heats upon consecutive additions of potassium chloride solution (247 mM) to BCETB in water (0.39 mM) at 298.15 K (symbols) and fits using two different binding models (solid lines): the sequential binding sites model (B) and the single set of identical sites model (C). The integrated heats are averages of the integrated heats from three replicas of the experiment, and the white bars within the symbols are the standard deviations. A heat flow diagram from one of the replicas is included in the top (A). The parameters predicted by each model are included as annotations, as well as the reduced chi-squared statistic, χ^2^/υ.

Figure 2 shows the heat flow diagram and the normalized, integrated heats obtained from the ITC experiments with potassium chloride (the corresponding experimental data and fits from addition of solutions of potassium iodide, potassium thiocyanate, and potassium sulfate to BCETB in water are reported in the Supporing Information). Two different binding models were employed: (i) the sequential binding sites model and (ii) the single set of identical sites model.^22^ In the former model (i), two binding constants are estimated; *K*_1_ defines the equilibrium between the states with zero and one potassium ion bound, whereas *K*_2_ corresponds to the binding of a second potassium ion. In the latter model (ii), only one binding constant, *K*, is defined, corresponding to the binding of a potassium ion to either of the sites regardless of whether there is another ion already bound to the other site.

To determine the goodness of fit, the reduced chi-squared statistic, χ^2^/υ, was calculated, which gives the ratio of the fitting error and the measurement error (see the Supporting Information). The sequential binding sites model fits the data significantly better than the single sites set of the identical sites model, as evident from the smaller value of χ^2^/υ for the former (χ^2^/υ = 12.02 compared to χ^2^/υ = 318.80). The introduction of more parameters in a model is associated with the risk of overfitting, which is implied by a value of χ^2^/υ significantly less than unity.^25^ This was not observed for either of the models, but due to the significantly smaller value of χ^2^/υ, we chose to describe the binding of potassium ions to BCETB using the sequential binding sites model. The corresponding fits applied to systems with three other potassium salts (KI, KSCN, and K_2_SO_4_) are reported in the Supporting Information.

The binding constants *K*_1_ and *K*_2_ obtained from the ITC experiments with BCETB for each of the potassium salts are presented in Table 1. In general, the estimated binding constants for the binding of the first potassium ion (*K*_1_) are similar to reported binding constants for the binding between 18-crown-6 and potassium (107 ± 25^26^ and 138 ± 7^27^). The binding constants for the binding of the second potassium ion (*K*_2_) are lower than would be expected for a ditopic receptor with two identical noninteracting binding sites. The binding constants describing the consecutive binding of several ligands to a receptor are related partly through statistical factors that depend on the total number of binding sites and the number of occupied binding sites (see the Supporting Information).^28,29^ For a receptor with two binding sites, this statistical factor gives that the binding constant for the binding of the second ligand should be four times lower than the binding of the first (referred to as statistical binding). If the relationship between the two binding constants deviates from this ratio, the binding sites are either not identical or behave cooperatively (i.e., the binding of one ligand influences the binding affinity for the second ligand). In our case, the binding constants for the binding of the second potassium ion (*K*_2_) are lower than the statistical binding, which indicates negative cooperativity. This is also expressed by the calculated stepwise cooperativity parameters (ρ, see Table 1), which are defined so that ρ < 1 means that the binding sites exhibit negative cooperativity.^23,24^

**Table 1:** Binding Constants^a^ and Cooperativity Parameters^b^ Obtained from ITC Experiments,^c^ Continuum Electrostatic Theory, and the MD Force Fields Using OPLS-AA, DFT Charges, and DFT Charges (pol.), the Latter Using Polarized Charges on the Complexes BCETB·K^+^ and K^+^·BCETB·K^+^

salt	method	*K*_1_	*K*_2_	ρ
KCl	ITC experiments	119 ± 9	17.2 ± 2.3	0.58 ± 0.09
	MD, OPLS-AA charges	41.6 ± 10.3	6.55 ± 1.63	0.63 ± 0.22
	MD, DFT charges	7000 ± 1700	700 ± 170	0.40 ± 0.14
	MD, DFT charges (pol.)	(19.6 ± 5.9)× 10^–3^	∼0 ± ∼0	∼0 ± ∼0
KI	ITC experiments	103 ± 6	15.6 ± 0.6	0.61 ± 0.04
	MD, DFT charges	2090 ± 380	385 ± 74	0.74 ± 0.20
KSCN	ITC experiments	139 ± 14	18.1 ± 1.7	0.52 ± 0.07
	MD, DFT charges	1900 ± 340	543 ± 87	1.14 ± 0.28
K_2_SO_4_	ITC experiments	133 ± 6	15.9 ± 1.4	0.48 ± 0.05

a The original binding constants in units of M^–1^ have been normalized with the standard concentration 1 M yielding the dimensionless binding constants, *K*_1_ and *K*_2_, making them directly related to the standard binding free energies through Δ*G*_bind_^°^ = −*RT* ln *K* (see Supporting Information).

b The stepwise cooperativity parameter is calculated according to ρ = 4β_2_/β_1_^2^, where β_1_ = *K*_1_ and β_2_ = *K*_1_*K*_2_.^23,24^

c The parameters estimated from ITC experiments are based on the normalized, integrated heats averaged over three replicas of each experiment. The associated errors are the standard deviations of the parameters generated with each replica.

The standard free energies and binding enthalpies estimated from the ITC experiments are shown in Table 2 and Table 3, respectively. While the binding of the first potassium ion has a more negative free energy than the second (ΔΔ*G*_bind_^°^ > 0), the binding of the second ion is, surprisingly, *enthalpically favored* (ΔΔ*H*_bind_^°^ < 0). In contrast to the rather small difference in the binding free energy of the first and second potassium ion (1.12–1.26 kcal/mol), the difference in their binding enthalpies is surprisingly large in magnitude, ranging from −2.64 to −5.27 kcal/mol, depending on the counterion. Part of the less favorable binding free energy for the second potassium ion can be attributed to the before-mentioned statistical factor (Figure S4, Supporting Information). At room temperature, this effect contributes with *RT* ln 4 ≈ 0.8 kcal/mol to ΔΔ*G*_bind_^°^. For the ITC experiments performed with potassium chloride, *T*ΔΔ*S*_bind_^°^ = ΔΔ*H*_bind_^°^ – ΔΔ*G*_bind_^°^ = −3.78 kcal/mol, meaning that the process of binding the second potassium ion is associated with an entropy decrease 3.78 kcal/mol larger than that of the first. Of these 3.78 kcal/mol, 0.8 kcal/mol is purely statistical, whereas the rest must be a consequence of additional, system-specific entropic losses upon binding (e.g., reduced flexibility of the complex or increased ordering of water in the solvation shells). This is in line with previous examples found in the literature, where negative cooperativity is usually found to be mainly entropy-driven (i.e., the binding of ligands results in the loss of configurational entropy).^30^

**Table 2:** Standard Binding Free Energies^a^ Obtained from ITC Experiments,^b^ Continuum Electrostatics, and the MD Force Fields Using OPLS-AA, DFT Charges, and DFT Charges (pol.), the Latter Using Polarized Charges on the BCETB and K^+^

salt	method	Δ*G*_bind,1_^°^ (kcal/mol)	Δ*G*_bind,2_^°^ (kcal/mol)	ΔΔ*G*_bind_^°^ (kcal/mol)
KCl	ITC experiments	–2.83 ± 0.05	–1.69 ± 0.08	1.14 ± 0.09
	MD, OPLS-AA charges	–2.2 ± 0.1	–1.1 ± 0.1	1.1 ± 0.1
	MD, DFT charges	–5.2 ± 0.1	–3.9 ± 0.1	1.4 ± 0.1
	MD, DFT charges (pol.)	2.3 ± 0.2	68.3 ± 0.2	66.0 ± 0.2
KI	ITC experiments	–2.74 ± 0.03	–1.63 ± 0.02	1.12 ± 0.04
	MD, DFT charges	–4.5 ± 0.1	–3.5 ± 0.1	1.0 ± 0.1
KSCN	ITC experiments	–2.92 ± 0.06	–1.72 ± 0.05	1.21 ± 0.08
	MD, DFT charges	–4.5 ± 0.1	–3.7 ± 0.1	0.7 ± 0.1
K_2_SO_4_	ITC experiments	–2.90 ± 0.03	–1.63 ± 0.05	1.26 ± 0.06
all	continuum electrostatics			0.37

a The difference in the standard binding free energies is calculated according to ΔΔ*G*_bind_^°^ = Δ*G*_bind,2_^°^ – Δ*G*_bind,1_^°^ with the exception of continuum electrostatic theory, where ΔΔ*G*_bind_^°^ = *G*_++_.

b The parameters estimated from ITC experiments are based on the normalized, integrated heats averaged over three replicas of each experiment. The associated errors are the standard deviations of the parameters generated with each replica.

**Table 3:** Standard Binding Enthalpies^a^ According to the ITC Experiment,^b^ Continuum Electrostatics, and the Force Fields Employing OPLS-AA and DFT Charges

salt	method	Δ*H*_bind,1_^°^ (kcal/mol)	Δ*H*_bind, 2_^°^ (kcal/mol)	ΔΔ*H*_bind_^°^ (kcal/mol)
KCl	ITC experiments	–4.12 ± 0.31	–6.76 ± 0.07	–2.64 ± 0.31
	MD, OPLS-AA charges	–4.1 ± 2.4	–4.7 ± 2.6	–0.6 ± 2.4
	MD, DFT charges	–3.1 ± 2.8	–4.8 ± 2.4	–1.7 ± 2.7
	MD, DFT charges (pol.)	8.5 ± 3.1	69.7 ± 2.6	61.2 ± 3.1
KI	ITC experiments	–4.54 ± 0.19	–7.35 ± 0.27	–2.81 ± 0.33
	MD, DFT charges	–1.8 ± 2.3	–2.2 ± 2.4	–0.5 ± 2.0
KSCN	ITC experiments	–3.61 ± 0.36	–8.88 ± 0.14	–5.27 ± 0.39
	MD, DFT charges	–3.7 ± 2.0	–4.4 ± 1.6	–0.8 ± 2.2
K_2_SO_4_	ITC experiments	–3.83 ± −0.14	–7.62 ± −0.30	–3.79 ± −0.34
all	continuum electrostatics			–0.14

a The difference in the standard binding enthalpies is calculated according to ΔΔ*H*_bind_^°^ = Δ*H*_bind, 2_^°^ – Δ*H*_bind,1_^°^ with the exception of continuum electrostatic theory, where ΔΔ*H*_bind_^°^ = *H*_++_.

b The parameters estimated from ITC experiments are based on the normalized, integrated heats averaged over three replicas of each experiment. The associated errors are the standard deviations of the parameters generated with each replica.

The trends that ΔΔ*G*_bind_^°^ > 0 and ΔΔ*H*_bind_^°^ < 0 are reproduced for all of the potassium salts (Tables 2 and 3), indicating that the observed thermodynamic trends are independent of the counterion. The absolute values of ΔΔ*G*_bind_^°^ are similar for all potassium salts. However, whereas potassium chloride, potassium iodide, and potassium sulfate show similar quantitative values of ΔΔ*H*_bind_^°^, the experiment with potassium thiocyanate shows an even more pronounced negative ΔΔ*H*_bind_^°^ (−5.27 kcal/mol compared to −2.64 kcal/mol for potassium chloride). One potential explanation for the observed differences could be the difference in hydration, where the thiocyanate ion is weakly hydrated in water and is known to possess a higher affinity for apolar surfaces compared to the more strongly hydrated chloride, iodide, and sulfate ions.^31,32^ The pronounced enrichment of thiocyanate ions around the receptor compared to the other counterions could be expected to stabilize the positive charge that is accumulated in the complex as the potassium ions bind to the receptor. However, to fully elucidate the effect of anion accumulation around BCETB on the magnitude of the negative ΔΔ*H*_bind_^°^, detailed analyses of where on BCETB the anions accumulate and of the type of interactions causing this accumulation are needed. Due to the poor convergence of such properties resulting from the low salt concentrations in the systems studied herein, these types of analyses were not feasible and are thus left for future studies.

Since the thermodynamic trends appear to be independent of the counterion, and we are mainly interested in the origin of the negative ΔΔ*H*_bind_^°^ value, the simulations and discussion presented below are mainly focused on the binding between BCETB and one of the salts, KCl.

### Continuum Electrostatic Theory

The consecutive binding of cationic metal ions to a single, ditopic receptor entails a contribution from the free energy of interaction between the cations, *G*_++_(*r*, *T*), limiting the stability of the final complex. Intuitively, this interaction is repulsive for any (finite) cation separation, *r*, and can be preliminarily estimated using Coulomb’s law, which for two monovalent ions of equal charge yields
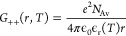
1where *e* is the elementary charge, *N*_Av_ is the Avogadro constant, ϵ_0_ is the vacuum permittivity, ϵ_r_ is the relative dielectric constant of the solvent, and *r* is the ion separation. The relative dielectric constant, ϵ_r_(*T*), contains all rotational and translational degrees of freedom of the solvent and is therefore a temperature-dependent quantity,^33^ whereby *G*_++_(*r*, *T*) should be regarded as a free energy. The enthalpy of interaction (for full derivation, see the Supporting Information) is hence given as
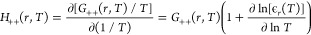
2

Water shows a particularly large, negative temperature response, and at *T* = 25 °C, ,^34^ whereby the enthalpy of interaction takes the opposite sign compared to the free energy of interaction: *H*_++_(*r*, 25 °C) = −0.37*G*_++_(*r*, 25 °C). The temperature derivative of the relative dielectric constant () is obtained from experiments^34^ and is thus an average measure of how the dielectric properties of water change with temperature. Hence, the model captures the temperature dependence of the water-mediated electrostatic interaction between two charges in the solution bulk.

*G*_++_(⟨*r*⟩) and *H*_++_(⟨*r*⟩), calculated according to eqs 1 and 2 with ⟨*r*⟩ = 11.5 Å (the average distance between the potassium ions in BCETB as determined in the simulations), yields the same signs as the corresponding experimental values (ΔΔ*G*_bind_^°^ > 0 and ΔΔ*H*_bind_^°^ < 0, Table 2 and Table 3). However, the magnitudes of the thermodynamic parameters are underestimated compared to the experimentally estimated values. The positive sign of ΔΔ*G*_bind_^°^ (*G*_++_(⟨*r*⟩)) is due to the entropic penalty of bringing the two potassium ions closer (from infinite separation to the average distance between the binding sites in BCETB). The model further correctly predicts a negative, however small, enthalpic contribution (*H*_++_(⟨*r*⟩) = ΔΔ*H*_bind_^°^ = −0.14 kcal/mol) to the difference in the binding free energies. The negative sign is a pure consequence of the dielectric property of water (), and the behavior is thus only observed in solvents where this is fulfilled (*in vacuo*, ϵ_r_ = 1 and  and in some solvents with a less pronounced dielectric temperature response, ). Continuum electrostatics also predict a *decrease* in entropy as the two cations approach each other: – *T*ΔΔ*S*_bind_^°^ = ΔΔ*G*_bind_^°^ – ΔΔ*H*_bind_^°^ = 0.51 kcal/mol (from Table 2 and Table 3). Although small, the enthalpy and entropy changes upon bringing the potassium ions from infinite separation (or bulk) to their average separation in BCETB (ΔΔ*H*_bind_^°^ = −0.14 kcal/mol and – *T*ΔΔ*S*_bind_^°^ = 0.51 kcal/mol) can explain parts of the experimentally estimated values (for KCl, ΔΔ*H*_bind_^°^ = −2.64 kcal/mol and −*T*ΔΔ*S*_bind_^°^ = 3.78 kcal/mol)

### Replica Exchange Molecular Dynamics

To gain a more detailed, molecular understanding of the mechanisms underlying the binding thermodynamics, we performed REMD simulations. Due to the lack of reliable force fields for divalent ions such as the sulfate ion, we included only the monovalent ions (chloride, iodide, and thiocyanate) in the simulation studies. For the system with BCETB and potassium chloride, we studied the conformational dynamics of BCETB, which enabled us to investigate the role of BCETB in the binding process. In addition, we have analyzed the water in the vicinity of the binding sites to elucidate its structural response to the binding of K^+^. Finally, to gain insight into the impact of the counterion, we have computed the relative affinities of chloride, iodide, and thiocyanate to BCETB.

For the purpose of the REMD simulations, starting from the OPLS-AA force field,^35^ we developed two different force fields, one using partial charges on BCETB according to the assigned OPLS-AA atom types and the other using charges determined from density functional theory (DFT)^36^ calculations (Table S2, Supporting Information). By employing these two different force fields, we enable a more robust comparison between experiments and simulations while simultaneously probing the impact of the partial charges on BCETB on the determined binding free energies and enthalpies. The force fields were validated by comparing the differences ΔΔ*G*_bind_^°^ = Δ*G*_bind,2_^°^ – Δ*G*_bind,1_^°^ and ΔΔ*H*_bind_^°^ = Δ*H*_bind,2_^°^ – Δ*H*_bind,1_^°^ obtained from simulation with those estimated from ITC experiments, with emphasis on the agreement in the latter. The best-performing force field was subsequently used to analyze changes in the conformational ensemble of BCETB and the solvent response upon binding of the first and second potassium ion. In addition, we tested a force field where the charges on BCETB were determined from DFT calculations, but for the bound states the charges for the bound potassium ion(s) and BCETB were determined simultaneously. This approach allows for charge transfer between the BCETB and bound potassium ion(s) and gives three sets of charges for the different complexes: the free BCETB, BCETB with one potassium ion bound, and BCETB with two potassium ions bound. By employing these charges in REMD simulations, we tested the effect of polarization of the complex upon the sequential binding of two potassium ions.

**Figure 3:**
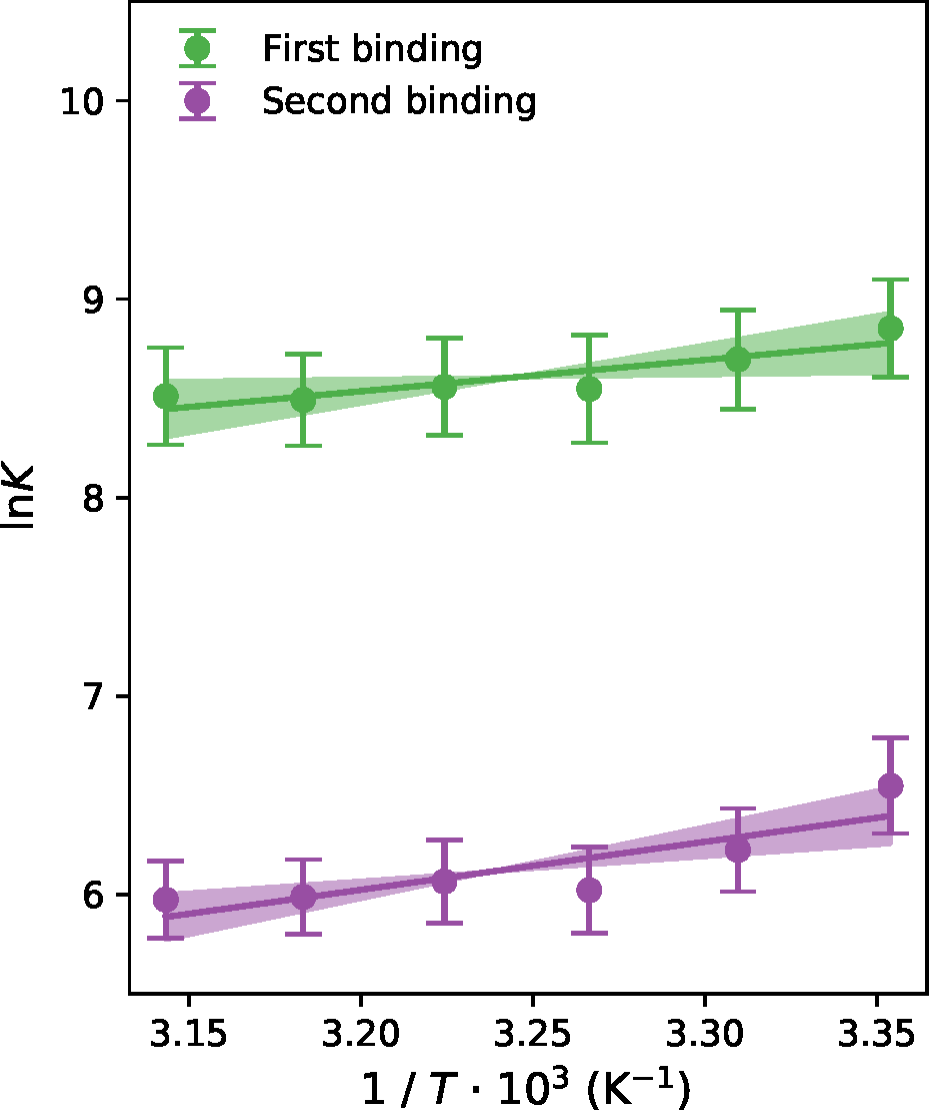
Standard binding free energies as a function of temperature obtained with DFT charges from REMD simulations with potassium chloride. The symbols are simulated points, the lines are linear fits used to calculate the standard binding enthalpies, and the shaded areas are the standard errors of the slopes, yielding the error estimates for the binding enthalpies. Green and purple color show the results obtained for the first and second binding event, respectively.

In Figure 3, simulated values of ln *K* = −Δ*G*_bind,*i*_^°^/(*RT*) for the system with potassium chloride, obtained with the force field using DFT charges, are plotted against 1/*T*, where *i* denotes the number in the series of the two binding events and the green and purple colors correspond to *i* = 1 (first K^+^) and *i* = 2 (second K^+^), respectively. To account for the effect related to the difference in the degree of degeneracy for the states with zero, one, and two K^+^ bound, these free energies have been corrected with the factors *RT* ln 1/2 and *RT* ln 2, respectively, to obtain the standard binding free energies. For the relative standard binding free energy (ΔΔ*G*°), this degeneracy effect contributes with a factor *RT* ln 4 (see Supporting Information). The slope of the linear least-squares fit obtained gives −Δ*H*_bind,*i*_^°^/*R* according to the linear van’t Hoff equation.^37^

The binding constants, standard binding free energies, and standard binding enthalpies determined from the REMD simulations are presented in Tables 1–3. For the system containing BCETB and potassium chloride, the thermodynamic parameters were determined using each of the three different force fields: OPLS-AA, DFT charges, and DFT charges (pol.). In general, both the force field using OPLS-AA and DFT charges reproduce the experimentally estimated trends in the thermodynamic parameters (Δ*G*_bind,1_^°^, Δ*G*_bind,2_^°^, Δ*H*_bind,1_^°^, Δ*H*_bind,2_^°^, and ΔΔ*H*_bind_^°^ < 0, whereas ΔΔ*G*_bind_^°^ > 0, Tables 2 and 3). For the force field using polarized DFT charges on BCETB (DFT charges (pol.)), the agreement with experimental values is poor, with inversed signs for all thermodynamic parameters except ΔΔ*G*_bind_^°^, which in turn is significantly overestimated (66.0 kcal/mol compared to 1.14 kcal/mol from experiments, Table 2). Hence, we chose to exclude this force field in further analysis and discussion. The inversed signs of the binding free energies and enthalpies might be a result of the net positive charge accumulated in BCETB during the charge transfer from the bound potassium ions (Table S3, Supporting Information), introducing repulsion between the receptor and bound ions. This is also reflected in the large binding site volume determined from the mean-squared positional fluctuation of the potassium ion in the binding site (Table S5, Supporting Information). We anticipate that to more correctly include the effect of polarization upon binding, DFT charges on the free BCETB and the complexes with one and two potassium ions bound determined in the presence of explicit water could be used.

For the other simulated salts (KI and KSCN), only the force field using DFT charges was employed due to its better performance in determining ΔΔ*H*_bind_^°^ for KCl (−1.7 kcal/mol compared to −0.6 kcal/mol from the force field using OPLS-AA charges, Table 3). For all of the three tested force fields, the binding free energy predictions are associated with fairly low standard deviations (0.1–0.2 kcal/mol), whereas the binding enthalpies show higher uncertainties (1.6–3.1 kcal/mol), resulting from the amplification of errors when performing the least-squares fit to obtain Δ*H*_bind,*i*_^°^ from the slope (Figure 3). The enthalpy predictions from simulation should thus be treated as qualitative rather than a quantitative predictions. As seen in Table 3, although the values of Δ*H*_bind,1_^°^, Δ*H*_bind,2_^°^, and ΔΔ*H*_bind_^°^ determined from the force field using DFT charges are underestimated compared to our experiments, the experimental trends are reproduced in terms of correct signs and orders of magnitude, for all simulated salts.

### Perturbation in Conformational Space

It has previously been shown that 18-crown-6 itself predominantly adopts four distinct conformations.^38^ To elucidate the conformational dynamics of BCETB, we performed principal component analyses (PCA) on the simulated conformations of the free BCETB and the complexes with one and two potassium ions obtained with the force field using DFT charges.

Resulting from the PCAs performed on the simulated conformations of BCETB in the system with potassium chloride, four well-defined conformations separated by notable energy barriers are distinguished. These conformations are characterized by having the CEs either bent out from the cavity or in toward the cavity (Figure S11). One of the conformations is more extended with both CEs bent out (EXT), whereas another appears collapsed with both CEs bent in (COL). The two other conformations appear as skewed, with one CE bent out and one bent in (SK1 and SK2). Due to the *C*_2_ symmetry of BCETB, the SK1 and SK2 conformations are identical (and hence the same conformation) for the free BCETB and the complex with two potassium ions bound. However, for the complex with one potassium ion bound, the *C*_2_ symmetry is broken. In this case, SK1 is defined as the conformation where the potassium ion is bound to the CE that is bent out, and SK2 is defined as the conformation where the potassium ion is bound to the CE that is bent in toward the cavity.

**Figure 4:**
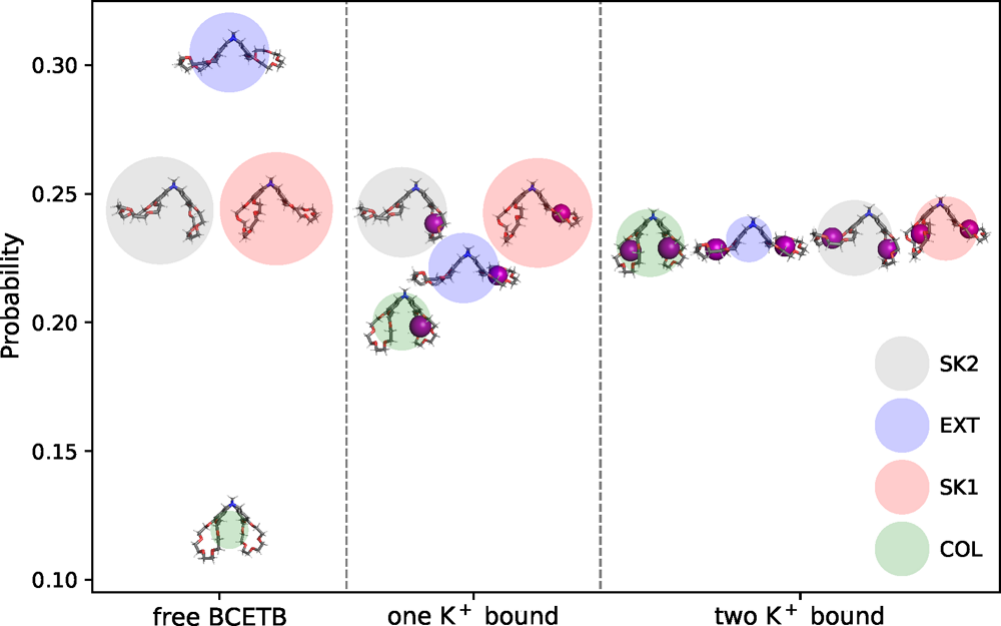
Integrated probabilities for the conformations in the vicinity of the four free energy minima (SK2 (gray), EXT (blue), SK1 (red), and COL (green)) obtained using DFT charges for the free BCETB and for complexes with one and two potassium ions bound (left, middle, and right) in the system with potassium chloride. The radius of each sphere equals the standard error of the corresponding probability.

The probabilities for each conformation (*p*_EXT_, *p*_COL_, *p*_SK1_, and *p*_SK2_) were obtained by integrating the sampled points in the PCA space within each of the drawn ellipses enclosing the minima (Figure S10). These probabilities are illustrated in Figure 4, showing that with no potassium ions bound, BCETB adopts the EXT conformation with highest probability, whereas the COL conformation exhibits the lowest probability among the four conformations. The conformations with one CE bent in and the other bent out (SK1 and SK2) exhibit intermediate and overlapping probabilities. Due to the *C*_2_ symmetry of BCETB, this is expected in the case of the free BCETB and the complex with two potassium ions bound. Surprisingly, the probabilities of the SK1 and SK2 conformations are found to be equal also for the complex with one potassium ion bound. Any difference in the solvation free energy of these conformations would favor one of them. On the other hand, the binding site separation is equal for these conformations, which implies that the interaction between the binding sites is not altered much when the complex switches from the SK1 to the SK2 conformation. The probability of the COL conformation increases successively upon binding of the first and second potassium ion. The stabilization of the conformation indicates that there is some favorable interaction introduced when the ions bind, either between water and BCETB in its collapsed state (i.e., increased solvation of this conformation upon binding) or between the binding sites.

The relatively large magnitudes of the experimentally estimated and the simulated binding enthalpies (Table 3) indicate strong attractive interactions between the potassium ion and the CE moiety of BCETB. It is thus plausible that the increased probability of the collapsed state is due to additional, stabilizing interactions introduced between the bound potassium ion and the opposite, free CE when the binding sites come closer. The additional increase of the probability of the collapsed conformer when the second K^+^ binds indicates further stabilizing interactions between the two CE·K^+^ groups. This is in line with the prediction from continuum electrostatics, and the seemingly attractive CE·K^+^–CE·K^+^ interaction could be responsible for an additional contribution to the large negative ΔΔ*H*_bind_^°^ observed in the ITC experiments. Even though the predictions of ΔΔ*H*_bind_^°^ from simulation show large overlapping errors (Table 3) and should be evaluated with care, the force field using DFT charges predicts a more negative ΔΔ*H*_bind_^°^ compared to the force field using OPLS-AA charges, and a plausible explanation could be the more favorable CE·K^+^–CE·K^+^ interactions depicted by the former. The force field using DFT charges entails (on average) more negative charges on the oxygens in the CEs, which in turn can explain the more favorable CE·K^+^–CE·K^+^ interactions.

### Internal Energies of the Complexes and the Conformations

Any enthalpy change observed in experiments is related to the internal energy change of the studied system, Δ*U*_sys_, through Δ*H*_sys_ = Δ*U*_sys_ + Δ(*PV*), where *P* and *V* are the pressure and volume of the system, respectively. From the PCA analysis, it is apparent that the binding of potassium ions to BCETB favors the COL conformation, whereas the probability of the EXT conformation decreases upon binding of the first potassium ion (Figure 4). To isolate the contribution from internal energies within the BCETB complexes (*U*_0_, *U*_1_, and *U*_2_ for the free BCETB and the complexes with one and two potassium ions bound, respectively) to ΔΔ*H*°, we have analyzed the changes in internal potential energies of the complexes upon coordinating a first and a second potassium ion. The internal potential energies are calculated as average energies of the simulated configurations generated in the REMD simulations, excluding water and counterions. These energies thus include the bonded and nonbonded interactions within BCETB (including the interaction between the binding sites) and the nonbonded interactions between BCETB and bound potassium ions. The bonded interactions include harmonic bonds, angular potentials, and dihedral potentials in BCETB, whereas the nonbonded potentials include Coulomb electrostatics and Lennard-Jones interactions, as defined in the simulations.

**Figure 5:**
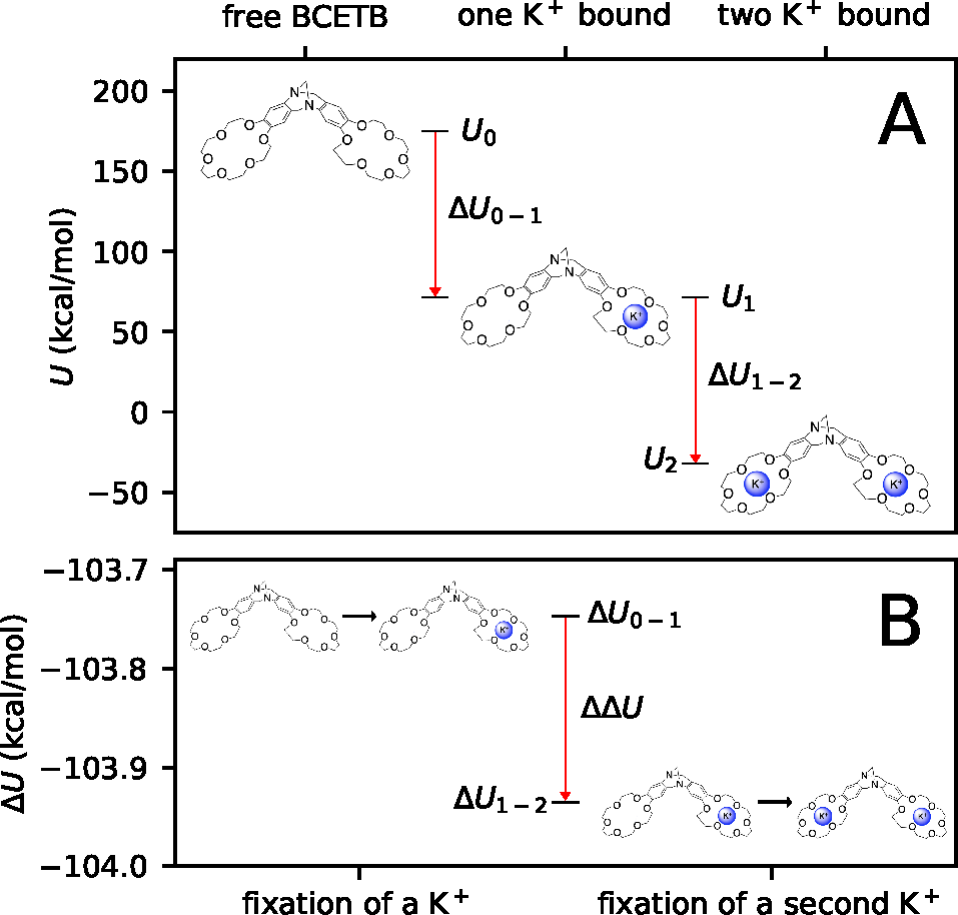
Internal potential energies of the complexes alone (water and chloride counterions excluded) averaged over all conformations using DFT charges with zero, one, and two potassium ions bound (A) and the differences in energy upon fixation of a first and a second potassium ion (B).

In Figure 5, internal potential energies (*U*_0_, *U*_1_, and *U*_2_) of the complexes with no, one, and two potassium ions bound using DFT charges are presented. The top plot (A) shows that both the energy of adding a first potassium ion (Δ*U*_0–1_ = *U*_1_ – *U*_0_) and the energy of adding a second potassium ion (Δ*U*_1–2_ = *U*_2_ – *U*_1_) are negative with similar magnitude. The relatively large magnitude (∼100 kcal/mol) is a result of the strong, attractive CE–K^+^ interactions formed when introducing a potassium ion in the binding site. In the bottom plot (Figure 5B), Δ*U*_0–1_ and Δ*U*_1–2_ are plotted together, showing the difference in the change in internal energy upon fixation of a second potassium ion compared to that of the first (ΔΔ*U* = Δ*U*_1–2_ – Δ*U*_0–1_). This can be interpreted as a measure of the contribution to the potential energy solely from the interaction between the sites, CE·K^+^–CE·K^+^, and the negative ΔΔ*U* (Table 4) indicates that favorable interactions between the sites are contributing to the overall negative ΔΔ*H*° that is observed in both experiment and simulations (Table 3). While the same result is predicted by continuum electrostatics (*H*_++_ < 0) where the enthalpic stabilization is a result of the dielectric properties of water () and thus is a solvent effect, we here exclude the water and thus calculate the interaction energy within the complex *in vacuo*. The enthalpy of interaction between two positive charges *in vacuo* is positive, and thus the negative sign of ΔΔ*U* must be a result of attractive interactions within the complex, overcompensating for the electrostatic ion–ion repulsion.

**Table 4:** Internal Potential Energy Changes of the Complex upon Binding of a First and a Second K^+^, Δ*U*_0–1_ and Δ*U*_1–2_, and the Difference between Those, ΔΔ*U* = Δ*U*_1–2_ – Δ*U*_0–1_, Using DFT Charges

force field	Δ*U*_0–1_ (kcal/mol)	Δ*U*_1–2_ (kcal/mol)	ΔΔ*U* (kcal/mol)
DFT charges	–103.75 ± 0.07	–103.94 ± 0.07	–0.19 ± 0.13

**Figure 6:**
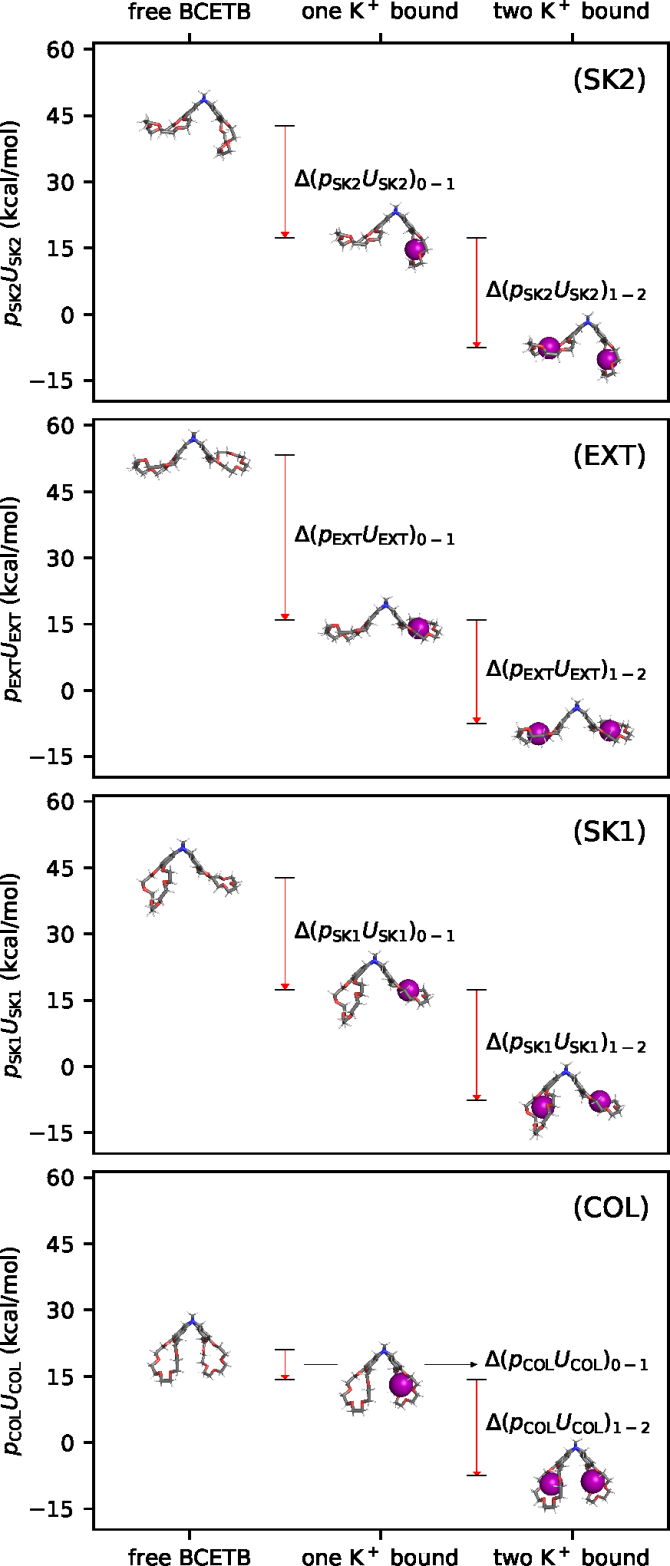
Measures of the probability-weighted potential energy, *pU*, for each conformation with zero, one, and two potassium ions bound using DFT charges for the system with potassium chloride. The differences in *pU* upon binding of a first and a second K^+^ are plotted as arrows. The annotations within parentheses included in each plot indicate the conformation.

To understand the origin of the negative ΔΔ*U*, we decomposed it into contributions from the four different conformations (SK2, EXT, SK1, and COL). Figure 6 shows the product *p*_*j*_*U*_*j*_ for the different conformations with no, one, and two bound K^+^ obtained using DFT charges, where *p*_*j*_ is the probability of conformation *j* obtained from the PCA analysis and *U*_*j*_ is the average internal potential energy of conformation *j*. In this way, the internal potential energy of each conformation and bound state is weighted with its corresponding probability in order to quantify its contribution to the internal potential energy of that bound state: *U*_*i*_ = ∑_*j*_*p*_*i,j*_*U*_*i,j*_, where *i* denotes the bound state (free BCETB, one K^+^ bound, or two K^+^ bound) and *j* denotes the conformation (SK2, EXT, SK1, COL, or the rest). In the same way, the contributions to Δ*U*_0–1_, *ΔU*_1–2_, and ΔΔ*U* were calculated by expressing them as the sums ∑_*j*_ Δ(*p*_*j*_*U*_*j*_)_0–1_, ∑_*j*_ Δ(*p*_*j*_*U*_*j*_)_1–2_, and ∑_*j*_ ΔΔ(*p*_*j*_*U*_*j*_), respectively. Figure 6 shows that the product *p*_*j*_*U*_*j*_ decreases upon binding of both a first and a second potassium ion for all conformations *j*. However, the magnitude of the decrease differs depending on conformation and bound state. The largest differences are observed for the EXT and COL conformations, where the former shows Δ(*p*_EXT_*U*_EXT_)_1–2_ > Δ(*p*_EXT_*U*_EXT_)_0–1_, whereas the latter shows the opposite: Δ(*p*_COL_*U*_COL_)_1–2_ < Δ(*p*_COL_*U*_COL_)_0–1_.

**Figure 7:**
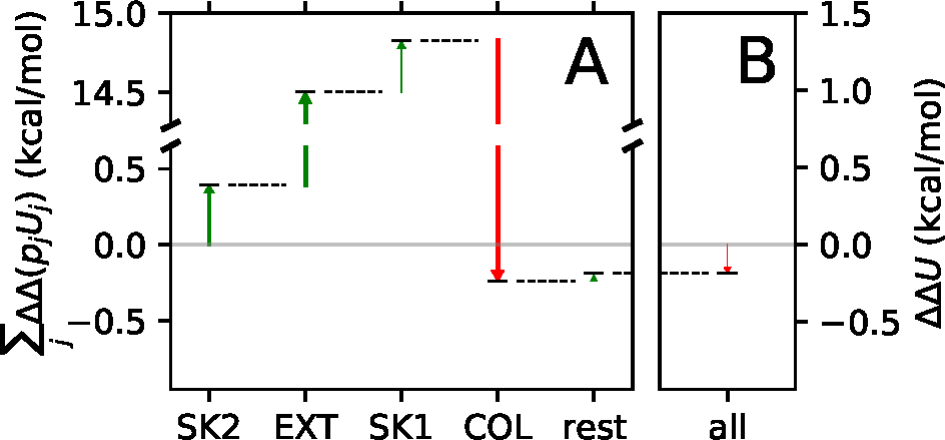
Running sum of contributions ΔΔ(*p*_*j*_*U*_*j*_) from the different conformations *j* to ΔΔ*U* (A) and ΔΔ*U* (B) using DFT charges. Red and green arrows indicate negative and positive contributions, respectively, and the width of each arrow has been scaled with the magnitude of the contribution for illustrative purposes. Note the broken *y*-axis in plot A.

The differences observed between the different conformations were quantified by calculating ΔΔ(*p*_*j*_*U*_*j*_) = Δ(*p*_*j*_*U*_*j*_)_1–2_ – Δ(*p*_*j*_*U*_*j*_)_0–1_. These are depicted as arrows in Figure 7A, and the running sum, ∑_*j*_ ΔΔ(*p*_*j*_*U*_*j*_), is the sum of arrows as a function of the conformations accounted for. All conformations except the COL conformation yield positive contributions to ΔΔ*U* (indicated by green arrows), and the EXT conformation shows a particularly large contribution (ΔΔ(*p*_EXT_*U*_EXT_) = 14.1 kcal/mol). On the contrary, the COL conformation is responsible for a large *negative* contribution (ΔΔ(*p*_COL_*U*_COL_) = −15.1 kcal/mol), which overcompensates for all of the positive contributions from the other conformations and makes ΔΔU negative (Figure 7B).

### Solvent Response

As previously discussed, continuum electrostatics predict that the presence of solvent results in an enthalpic attraction between two cations in water. To gain further insight into the effect of solvent reorganization upon binding, we have analyzed the solvation shell correlations between the two binding sites.

**Figure 8:**
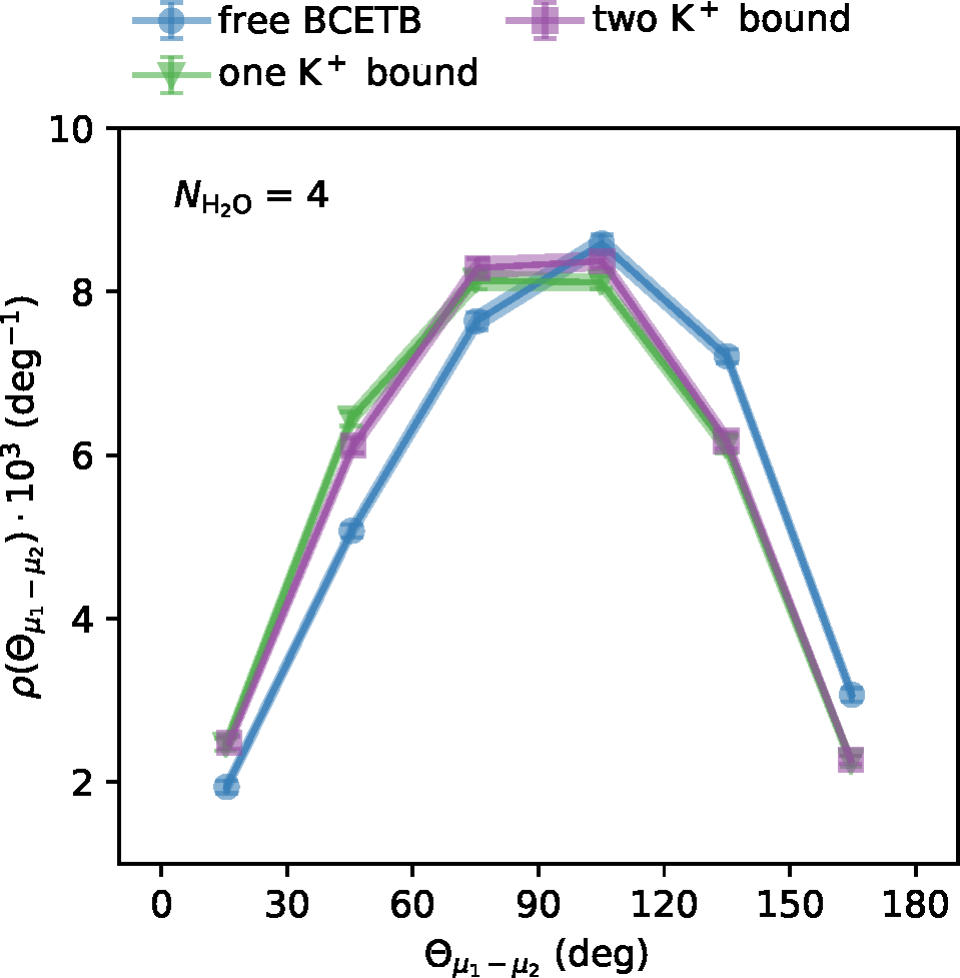
Probability density of angles between the dipoles of two water clusters, one at each binding site, using DFT charges for the system with potassium chloride. Each cluster is defined as the four closest water molecules to the binding site. The range of angles on the *x*-axis has been binned into subranges, where each marker represents the probability density within angles ±15° from the *x*-position of the marker (e.g., the leftmost markers show the probability densities of angles between 0° and 30°). The lines connecting the markers are merely guides, and the shaded areas show the interpolated errors between the probability densities for each subrange.

Figure 8 shows the distributions of angles between the dipole moment of water molecules in the solvation shells around each binding site, calculated using the force field with DFT charges. The clusters are defined as the four closest water molecules around each binding site. Some changes can be identified; upon binding of the first potassium ion, the distribution is slightly shifted toward smaller angles between the dipoles of the water clusters. However, these changes are subtle and the average angle between the dipoles varies only little (96°, 89°, and 90° for the states with no K^+^, one K^+^, and two K^+^ bound, respectively). Thus, alignment of the solvation water around the two sites upon binding of the first potassium ion is likely a minor contribution to the enthalpically more favorable binding of the second potassium ion. We performed the same analysis on larger clusters (8 and 12 water molecules), again with subtle correlations between the two solvation shells (see Figure S12 in the Supporting Information). This analysis is congruent with the fact that the negative ΔΔ*H*_++_(*r*) predicted by continuum electrostatics is small. The small contribution is expected since the average distance between the sites in BCETB, ⟨*r*⟩ = 11.5 Å, is significantly larger than the Bjerrum length, λ_B_, which is the distance at which the electrostatic energy between two charges is comparable to the thermal energy, *k*_B_*T*, where *k*_B_ is the Boltzmann constant (λ_B_ = *e*^2^/(4*πε*_0_ε_r_*k*_B_*T*) ≈ 7 Å for water at 25 °C).

### Affinity of Anions to BCETB

**Figure 9:**
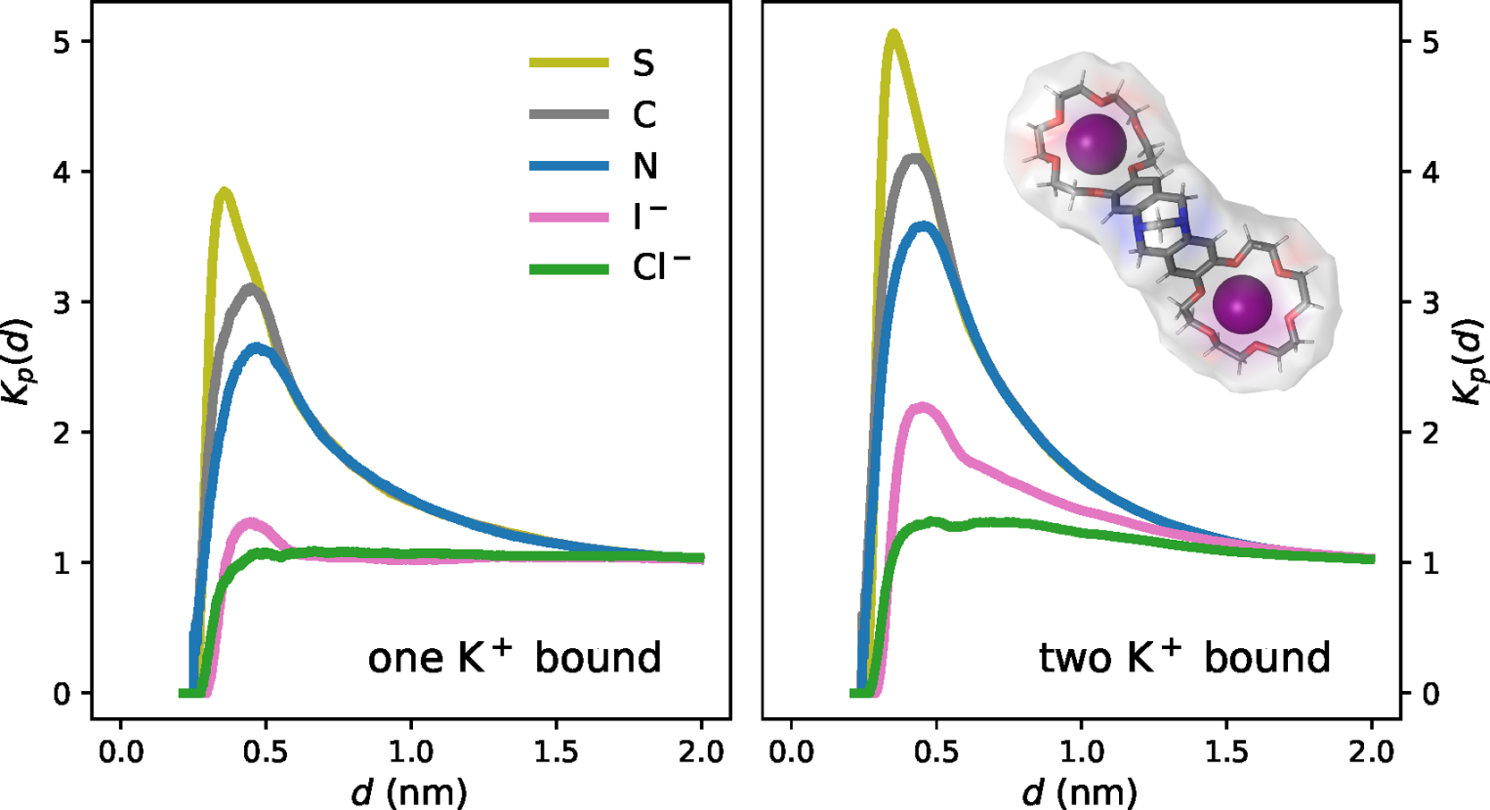
Local/bulk partition coefficients, *K*_p_(*d*), for chloride (Cl^–^), iodide (I^–^), and the atoms in thiocyanate (S, C, and N) as functions of the distance, *d*, to the BCETB surface. The inset shows an example of a volume *V*(*d*) around BCETB for which the cumulative ion/water radial distributions are calculated.

Experimentally, the system with potassium thiocyanate yielded a significantly more negative ΔΔ*H*_bind_^°^ value compared to the systems with the other potassium salts (Table 3). This indicates that there are specific counterion effects involved in the binding of KSCN to BCETB, where the thiocyanate anion enthalpically favors the binding of a second potassium ion compared to when chloride, iodide, or sulfate is the counterion. In order to further investigate this observation, the distributions of the counterions around the BCETB surface were analyzed. Figure 9 shows local/bulk partition coefficients of the different counterions with respect to the BCETB surface defined as^39^
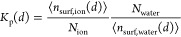
3Here, *d* is the distance from the BCETB surface, ⟨*n*_surf,ion_(*d*)⟩ and ⟨*n*_surf,water_(*d*)⟩ are the average numbers of counterions and water molecules, respectively, populating a region within [0, *d*] from the BCETB surface, whereas *N*_ion_ and *N*_water_ are the total numbers of counterions and water molecules, respectively, in the simulation box. For thiocyanate, we calculated *K*_p_(*d*) for the individual atoms separately, to get insight into the relative affinities of the sulfur, nitrogen, and carbon atoms to BCETB. For water, the oxygen atom was chosen as the reference atom. Figure 9 shows that while all counterions are enriched around BCETB compared to water, the atoms in thiocyanate accumulate more than chloride and iodide. The average distance from the atoms in thiocyanate to BCETB follows the order S < C < N, implying a preferential orientation where the sulfur atom points toward BCETB. This is in agreement with previously observed higher affinities of the thiocyanate ion to apolar surfaces compared to more strongly hydrated anions.^31,32^ As previously mentioned, the enrichment of thiocyanate ions around BCETB compared to the other counterions could contribute to the stabilization of the complex with one or two potassium ions bound.

## Conclusions

We have discovered counterintuitive thermodynamics governing the binding of potassium ions to a ditopic bis(18-crown-6) receptor. By means of ITC, we have found that the binding of a second potassium ion is enthalpically favored over that of the first, ΔΔ*H*_bind_^°^ < 0, despite the electrostatic repulsion one might expect is introduced upon binding of the second ion. The experimentally observed enthalpic stabilization is supported by continuum electrostatic theory involving the temperature derivative of the dielectric constant. The experimentally estimated positive ΔΔ*G*_bind_^°^ results from a much larger entropic penalty associated with the binding of a second K^+^ compared to that of a first, which compensates for the experimentally estimated negative ΔΔ*H*_bind_^°^. Comparison of the experimentally estimated binding constants (*K*_1_ and *K*_2_) also revealed negative cooperativity between the two binding sites in BCETB (ρ < 0).

The observed thermodynamic *trends* (ΔΔ*H*_bind_^°^ < 0 and ΔΔ*G*_bind_^°^ > 0) were found to be independent of the counterions investigated. However, thiocyanate further enhances the negative value of ΔΔ*H*_bind_^°^ compared to chloride, iodide, and sulfate ions. A possible explanation for this is the relatively higher affinity of the weakly hydrated thiocyanate ion to apolar surfaces compared to more hydrated anions.^31,32^ This was further supported by the analysis of the distribution of counterions around BCETB with one or two potassium ions bound, where we found a significant enrichment of thiocyanate close to BCETB compared to the other counterions.

Through the analysis of the REMD simulation trajectories using DFT-derived partial charges, we have provided further insight into the molecular mechanisms underlying the experimental observations. In particular, we have analyzed the conformational space of the free BCETB and its complexes with one and two potassium ions bound from simulations with potassium chloride, revealing four distinct conformations. The probability of the most compact conformation with both CEs bending inward (denoted COL) was found to increase successively upon binding of the first and second potassium ion. By analyzing the internal potential energies within the complexes with no, one, and two potassium ions bound (ΔΔ*U*), we found that these energies alone are responsible for a negative contribution to ΔΔ*H*_bind_^°^. By further weighting the individual internal potential energies of the different conformations with their respective probabilities, we found that the negative ΔΔ*U* is resulting primarily from the increasingly populated COL conformation upon binding, overcompensating for positive contributions to ΔΔ*U* from the rest of the conformations.

While previous studies have found the apparent attraction between metal cations bound to neutral receptors to primarily be a result of the decreased solvation free energy of the whole complex,^17−19^ we found only a subtle solvent response upon binding of the potassium ions to BCETB. Instead, we found that the specific interactions within the complex result in increased attraction between the binding sites upon binding of potassium ions, as manifested by the promotion of the COL conformation. The increased probability of the COL conformation upon binding was further found to result in a significant contribution to the negative ΔΔ*H*_bind_^°^ by calculating probability-weighted internal potential energies of the complexes. We anticipate that to further elucidate the origin of the pronounced enthalpic stabilization observed for the complex with two potassium ions bound, the role of the counterion in the binding process needs to be studied further. While we herein limited ourselves to only report the relative affinities of the counterions to BCETB, further details about the interactions causing thiocyanate to accumulate around BCETB could reveal counterion-specific contributions to the stabilities of the found principal conformations of the potassium–BCETB complexes.

In summary, in this study we have aimed to elucidate the different contributions to the overall interaction between potassium ions bound to a ditopic receptor. By studying the binding process from three different perspectives—experiment, continuum theory, and atomistic simulation—we have investigated the influence of different factors such as solvation, conformational changes, and the counterion. The discovered perturbation of the conformational ensemble of BCETB upon binding of one or two potassium ions provides insight into the role of the receptor in host–guest chemistry, and we anticipate that this work can be of importance for the design of synthetic receptors. From continuum electrostatics, the negative enthalpic contribution to the interaction between bound cations resulting from the dielectric temperature response of water is a fundamentally important result that can hopefully contribute to our understanding of electrostatic interactions between charged molecules in solution.

## Materials and Methods

### Materials

BCETB was synthesized following a previously reported procedure.^15^ Potassium chloride (99.0–100.5%) and potassium thiocyanate (99.0%) were purchased from Sigma-Aldrich. Potassium iodide (99.0%) was purchased from Acros Organics. Potassium sulfate (99.0%) was purchased from Merck. The salts were dried in an oven overnight prior to weighing. All solutions were prepared in volumetric flasks using deionized water.

### Isothermal Titration Calorimetry

The isothermal titration calorimetry experiments were performed using a MicroCal VP-ITC instrument having a cell volume of 1.4631 mL. Prior to each titration, the solutions of titrand and analyte were degassed for 5 min at 20 °C using a Thermovac instrument. The ITC experiments were performed at 25 °C, with 307 rpm stirring and the reference power set to 25 μcal/s. The titrations were performed by injecting 10 μL portions of the titrant ([K^+^] = 147–247 mM) into a 0.39–0.40 mM solution of BCETB, with a 300 s delay between each injection. An initial injection of 4 μL was discarded from each data set in order to remove the effect of the titrant diffusing across the syringe tip during the prerun equilibration process. Heats of dilution determined in the absence of receptor were subtracted from the titration data prior to curve fitting. Each titration experiment was performed in triplicate. The raw data were analyzed using Python 3, using both a one-site and sequential binding sites curve fitting model. Further details regarding the ITC experiments are given in the Supporting Information.

### Density Functional Theory

Partial charges on the atoms in BCETB were obtained using DFT^36^ calculations in Gaussian.^40^ To generate the input, BCETB was energy minimized in Avogadro, followed by minor adjustments of the atom positions to make the molecule *C*_2_ symmetric. The symmetric structure was used as input to Gaussian, followed by geometry optimization. From the optimized structure, charges were obtained by fitting to the electrostatic potential using the Merz–Singh–Kollman scheme,^41,42^ employing the B3LYP functional^43,44^ and the 6-31+G basis set. Partial charges were calculated for both the free BCETB and the complexes with one and two potassium ions bound in order to take into account polarization upon binding.

### Replica Exchange Molecular Dynamics

Replica exchange molecular dynamics simulations were performed using GROMACS 2019/4.^45^ Energies of the initial configurations were minimized using the steepest descent algorithm. From the minimized configurations, the system was equilibrated in two steps, and from the equilibrated configuration production runs were performed. For both equilibration and production runs, a leapfrog stochastic dynamics integrator^46^ was used, implicitly handling the temperature coupling, and the time step was set to 0.002 ps. In the first equilibration step, the system was run in the *NVT* ensemble for 20 ps, with an inverse friction constant of 1.0 ps^–1^ and a heat bath temperature of 298.15 K. In the second step, the system was equilibrated in the *NPT* ensemble for 1 ns using the Berendsen barostat^47^ with a relaxation time of 0.5 ps and a reference pressure of 1 bar. Production runs were performed in the *NPT* ensemble using the Parrinello–Rahman barostat,^48^ with a relaxation time of 1.0 ps and isothermal compressibility of 4.5 × 10^–5^ bar^–1^. For the solvation process, 30 ns production runs were performed, whereas the simulation time was extended to 50 ns for the complexation processes in order to sample enough of the different regions of the conformational space of BCETB. In all simulations, a cubic simulation box was used with initial dimensions of 4.0 × 4.0 × 4.0 nm^3^ (prior to *NPT* equilibration). For potassium, chloride, and iodide, we used charges and Lennard-Jones (LJ) parameters from the OPLS-AA force field.^49−52^ For thiocyanate, we used charges and LJ parameters from a recently developed force field.^32^ For BCETB, we applied LJ parameters according to the OPLS-AA force field, whereas the charges were assigned using three different approaches. In the first approach, we assigned partial charges according to the OPLS-AA force field, where the partial charges were equally shifted on all atoms in order to make the compound electroneutral. In the second approach, we used partial charges obtained from DFT calculations on the free BCETB. In the third approach, three sets of partial charges obtained from DFT calculations were used during the creation of the first and second potassium ion in the binding sites: the charges on the free BCETB and the charges on BCETB with one and two potassium ions bound, respectively (Figure S7 and Table S2 in the Supporting Information). The latter approach allowed us to include the effect of polarization of the complexes upon binding. All simulations were run using the SPC/E^53^ water model.

Due to the large conformational space of BCETB, replica exchange was utilized in an attempt to achieve more efficient sampling. Simulations were performed at six different temperatures in the range 298.15–318.15 K. The temperature spacing was chosen to be 4 K, which has previously been suggested in order to achieve optimal exchange probabilities in the *NPT* ensemble.^54^ The range of temperatures simulated also enabled the estimation of binding enthalpies. To obtain free energy differences, the GROMACS implementation of the Bennett acceptance ratio^55^ was used. Soft-core interactions were applied to both electrostatics and Lennard-Jones interactions to prevent the system from having overlapping particles as it is decoupled, with a soft-core α of 0.5, a soft-core power of 1.0, and a soft-core σ of 0.3 nm. In addition to the fully decoupled and the fully coupled state, 31 intermediate states were simulated in order to make the histograms of acceptance probabilities overlapping and reduce the errors to a satisfactorily degree.

For the analyses of the conformational space of BCETB, internal potential energies within the complexes, solvent response, and counterion affinity, longer simulations of 100 ns were performed.

## Supporting Information Available

ITC experiments with additional potassium salts, validation of experimental method, continuum electrostatic theory, correction factors due to the standard state and binding to ditopic receptors, computational details, Van’t Hoff plots, PCA analysis, and solvent response (PDF)

## Notes

The simulation results and setup, together with the experimental data analysis are available in an electronic notebook (Jupyter) at https://doi.org/10.5281/zenodo.6008299.
