# Supplementary material for: Counterintuitive Electrostatics upon Metal Ion Coordination to a Receptor with Two Homotopic Binding Sites
Source: J Am Chem Soc. 2022 Feb 10;144(7):2921–32. doi: 10.1021/jacs.1c08507 (PMC8874967; doi:10.1021/jacs.1c08507)
Supplement: Supplementary file 1 — ja1c08507_si_001.pdf [file ja1c08507_si_001.pdf]

Supporting Information: Counter Intuitive Electrostatics upon Metal Ion Coordination to a Receptor with Two Homotopic Binding Sites

Vidar Aspelin,† Anna Lidskog,‡ Carlos Solano Arribas,‡ Stefan Hervø-Hansen,† Björn Stenqvist,¶ Richard Chudoba,† Kenneth Wärnmark,‡,‖ and Mikael Lund†,§,‖

†*Division of Theoretical Chemistry, Department of Chemistry, Lund University, Lund SE 221 00, Sweden.*

‡*Center for Analysis and Synthesis (CAS), Department of Chemistry, Lund University, Lund SE 221 00, Sweden.*

¶*Division of Physical Chemistry, Department of Chemistry, Lund University, Lund SE 221 00, Sweden.*

§*Lund Institute of Advanced Neutron and X-ray Science (LINXS), Scheelevägen 19, Lund SE 223 70, Sweden.*

‖*K.W. and M.L. contributed equally to this work*

Isothermal titration calorimetry

Figure S1 shows the results from the ITC experiments with BCETB and potassium iodide, potassium thiocyanate, and potassium sulfate, respectively. To generate the fitted curves and

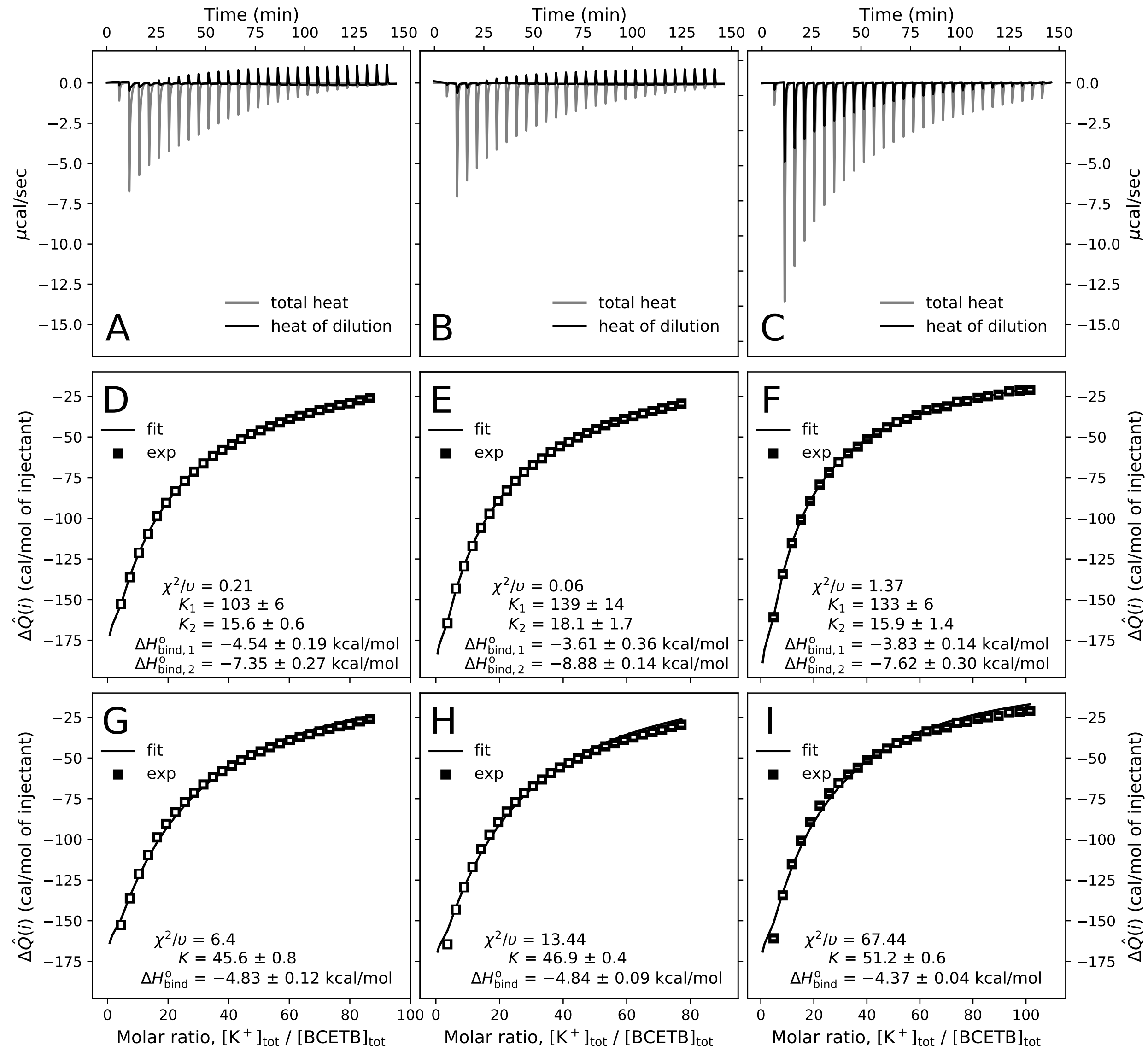

Figure S1: The left (A, D, and G), the middle (B, E, and H), and the right (C, F, and I) columns show results from ITC experiments where aqueous solutions of KI (209 mM), KSCN (147 mM), and K_2SO_4 (99 mM), respectively, were added to aqueous solutions of BCETB (0.39-0.40 mM). One heat flow diagram for each salt is included in the top row (A, B, and C). The symbols in the bottom two rows of plots show the integrated heats averaged over three replicas of each experiment, and the white bars within the symbols are the standard deviations. The middle (D, E, and F) and bottom (G, H, and I) rows show fits using the sequential binding sites model and the single set of independent binding sites model, respectively (solid lines). The parameters predicted by each model are included as annotations, as well as the reduced chi-squared statistics, χ^2/υ.

the associated parameters, we used Python 3 (the source code is included in the supporting information provided electronically). Two different models were employed, one assuming two identical, independent sites and the other assuming sequential binding.[S1] The quality of the fits were compared by calculating the reduced chi-squared statistic:

$$\frac{\chi^2}{\upsilon} = \frac{1}{\upsilon} \sum_{i=1}^{n} \frac{(y_i - f_i)^2}{\sigma_i^2} \tag{S1}$$

where y_i is the i'th measured heat, f_i is the corresponding prediction by the model, n is the number of measured heats, and $\upsilon = n - p$ is the number of degrees of freedom, where p is the number of parameters fitted in the model ($p = 2$ in the single set of independent binding sites model and $p = 4$ in the sequential binding sites model).

The sequential binding sites model results in significantly smaller values of χ^2/υ compared to the single set of independent binding sites model for all salts, indicating better agreement between the experimentally measured heats and the heats calculated in the model. However, for potassium iodide and potassium thiocyanate, the reduced chi-squared resulting from the sequential binding sites model is less than unity (Figure S1D and S1E), which either implies that the model is over-fitting the experimental data, or that the estimated measurement errors are overestimated. The error assigned to each heat was estimated by calculating the standard deviation among three replicas of the experiment. Due to the small number of replicas, it is not unlikely that these errors are overestimated. The overestimation of errors seems like a more plausible explanation than over-fitting, since we obtained $\chi^2/\upsilon > 1$ for both potassium chloride (Figure 2) and potassium sulfate (Figure S1F).

Validation of experimental method

Due to the low solubility of BCETB in water, the ITC experiments were performed using a relatively low concentration of the receptor (0.39 mM). In order to still ensure sufficient saturation of the receptor, higher concentrations of the salt solutions were used (147-247

mM). To validate the experimental setup, an ITC experiment was also performed with 18-crown-6 (0.36 mM) and KCl (100 mM). Figure S2 shows the heat flow diagram (A) and the normalized, integrated heats (B) from the ITC experiment. Since 18-crown-6 only contains one binding site, we used the single set of independent binding sites model for the fitting. The so-obtained thermodynamic parameters were compared with and show good agreement with two earlier studies (Table S1).[S2,S3]

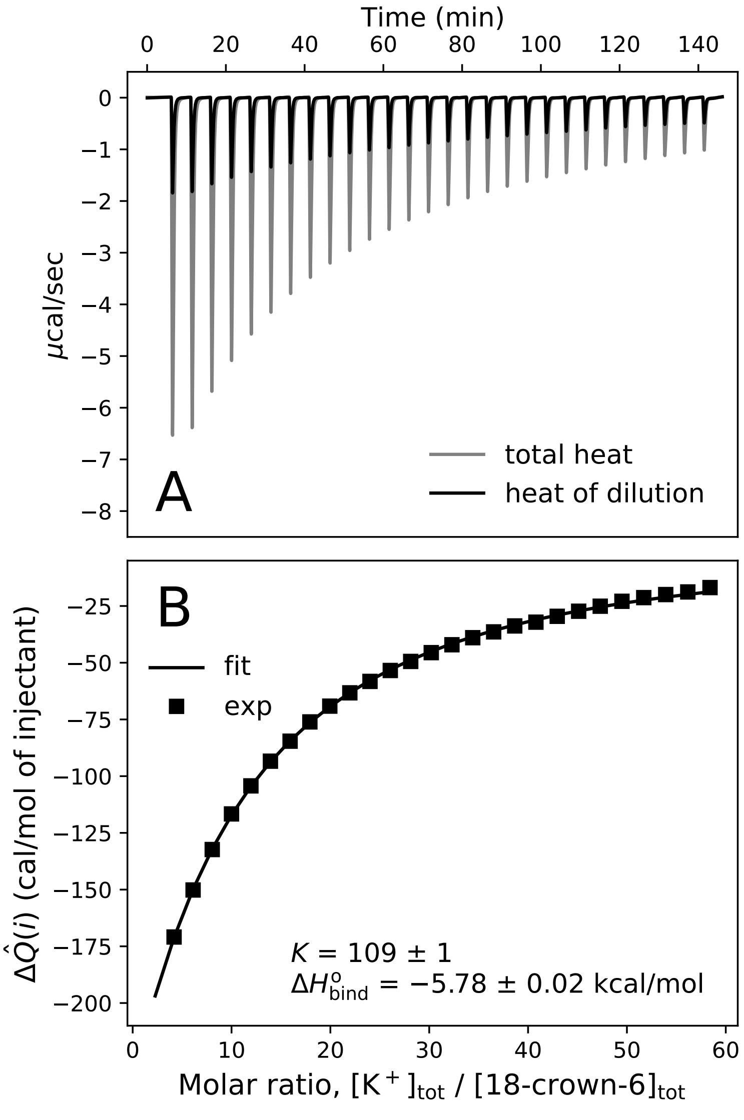

Figure S2: Heat flow diagram (A) and normalized, integrated heats (symbols, B) upon consecutive additions of potassium chloride solution (100 mM) to 18-crown-6 in water (0.36 mM) at 298.15 K. The fit (line, B) is obtained using the single set of independent binding sites model. The parameters predicted by the model are included as annotations.

Table S1: Binding constants[a] and enthalpies of binding of K^+ to 18-crown-6 in water at 298.15 K.

ref	K	$\Delta H^\circ_{\text{bind}}$ (kcal/mol)
this work[b]	109 ± 1	−5.78 ± 0.02
Izatt *et. al.*[S2]	107 ± 25	−6.21 ± 0.01
Michaux and Reisse[S3]	138 ± 7	−5.60 ± 0.20

[a] The original binding constant in units of M^{-1} have been normalized with the standard concentration 1 M yielding the dimensionless binding constant, K, making it directly related to the standard binding free energy through $\Delta G^\circ_{\text{bind}} = -RT \ln K$.

[b] Since we only performed one experiment with 18-crown-6, the errors on the parameters were estimated by performing non-parametric bootstrapping on the residuals from the least squares fitting.[S4]

Continuum model

The enthalpic contribution to the free energy of bringing two ions from infinite separation to a separation r is derived via the temperature derivative of the Coulomb free energy. The valencies are omitted for simplicity and since the valency of K^+ is +1, and we have:

$$\begin{aligned} H_{++} &= \frac{\partial(G_{++}/T)}{\partial(1/T)} = G_{++} + \frac{1}{T}\frac{\partial G_{++}}{\partial(1/T)} = G_{++} - T\frac{\partial G_{++}}{\partial T} = \frac{e^2 N_A}{4\pi\epsilon_0 r}\left(\frac{1}{\epsilon_r} - T\frac{\partial(\frac{1}{\epsilon_r})}{\partial T}\right) \\ &= \frac{e^2 N_A}{4\pi\epsilon_0 r}\left(\frac{1}{\epsilon_r} - T\frac{\partial(\frac{1}{\epsilon_r})}{\partial \epsilon_r}\frac{\partial \epsilon_r}{\partial T}\right) = \frac{e^2 N_A}{4\pi\epsilon_0 r}\left(\frac{1}{\epsilon_r} + \frac{T}{\epsilon_r^2}\frac{\partial \epsilon_r}{\partial T}\right) = G_{++}\left(1 + \frac{T}{\epsilon_r}\frac{\partial \epsilon_r}{\partial T}\right) \\ &= G_{++}\left(1 + \frac{\partial \ln \epsilon_r}{\partial \epsilon_r}\frac{\partial T}{\partial \ln T}\frac{\partial \epsilon_r}{\partial T}\right) = G_{++}\left(1 + \frac{\partial \ln \epsilon_r}{\partial \ln T}\right) \end{aligned} \tag{S2}$$

The only temperature dependent quantity is the dielectric constant, ϵ_r, and everything else is factored out. The entropic contribution is derived in a similar manner:

$$-TS_{++} = T\frac{\partial G_{++}}{\partial T} = -G_{++}\left(\frac{\partial \ln \epsilon_r}{\partial \ln T}\right) \tag{S3}$$

Binding free energies

The standard state

The association between two molecules, A and B, forming a complex, AB, is experimentally often quantified by determining the association constant, $\widetilde{K}^{\text{exp}}$. The association constant is obtained by measuring the activities of free and complexed molecules at equilibrium

$$\widetilde{K}^{\text{exp}} = \frac{a_{AB}}{a_A a_B} = \frac{c_{AB}\gamma_{AB}}{c_A\gamma_A c_B\gamma_B} = \frac{c_{AB}}{c_A c_B}\frac{\gamma_{AB}}{\gamma_A\gamma_B} \tag{S4}$$

where a_i, c_i, and γ_i are the activity, the concentration, and the activity coefficient of species i, respectively. If the number of molecules in the numerator is different from that in the denominator, $\widetilde{K}_{\text{exp}}$ will have units of concentration, and the resulting value depends on which concentration scale is used. Experimentally, $\widetilde{K}_{\text{exp}}$ is usually given in molar units.[S5] The association constant is related to the standard state binding free energy according to

$$\Delta G^{\circ}_{\text{bind}} = -RT\ln K \tag{S5}$$

However, this expression requires a dimensionless association constant, K, and to achieve this, a standard concentration, c°, needs to be added to Equation S4 according to

$$K^{\text{exp}} = \frac{c_{AB}c^{\circ}}{c_A c_B}\frac{\gamma_{AB}}{\gamma_A\gamma_B} = \widetilde{K}^{\text{exp}}c^{\circ} \tag{S6}$$

The standard concentration needs to have the same unit as the concentrations for the involved species, and is typically chosen to be 1 M. The experimental standard state free energy is thus obtained as

$$\Delta G^{\circ}_{\text{bind}} = -RT\ln K^{\text{exp}} = -RT\ln\left(\widetilde{K}^{\text{exp}}c^{\circ}\right) \tag{S7}$$

When comparing simulated and experimental binding free energies, they have to refer to

the same standard state in order for the comparison to be meaningful. In simulations, the concentration is typically not the standard concentration. In fact, the only concentration accessible in the simulation is the concentration of species one prepares the system with, and if the freedom of the involved species is not restricted by anything else than the volume of the box, any free energy prediction refers to this concentration. The relation between this computed free energy and the corresponding association constant is thus

$$\begin{aligned}\Delta G_{\text{bind}}^{\text{sim}} &= -RT\ln K^{\text{sim}} \\ &= -RT\ln\left(\frac{c_{AB}c^{\text{sim}}}{c_A c_B}\frac{\gamma_{AB}}{\gamma_A\gamma_B}\right)\end{aligned} \tag{S8}$$

where c^{sim} is the concentration of the binding substrate in the simulation. In principle, any concentration could be chosen to be the standard state, and if the concentration of the binding substrate indeed was chosen as the standard state so that $c^{\text{sim}} = c^{\circ}$, $\Delta G_{\text{bind}}^{\text{sim}}$ could be rewritten as $\Delta G_{\text{bind}}^{\circ}$. However, if the standard state applied to experimental results entails a different concentration than that used in simulations (which is usually the case), one has to correct the binding free energy obtained from simulation according to

$$\begin{aligned}\Delta G_{\text{bind}}^{\circ} &= -RT\ln\left(\frac{c_{AB}c^{\circ}}{c_A c_B}\frac{\gamma_{AB}}{\gamma_A\gamma_B}\right) \\ &= -RT\ln\left(K^{\text{sim}}\frac{c^{\circ}}{c^{\text{sim}}}\right) \\ &= \Delta G_{\text{bind}}^{\text{sim}} - RT\ln\left(\frac{c^{\circ}}{c^{\text{sim}}}\right)\end{aligned} \tag{S9}$$

Equation S9 can also be written in terms of molar volumes instead of concentrations, resulting in

$$\Delta G_{\text{bind}}^{\text{o}} = \Delta G_{\text{bind}}^{\text{sim}} + RT\ln\left(\frac{V^{\circ}}{V^{\text{sim}}}\right) \tag{S10}$$

where V^{sim} is the molar volume of the binding substrate in the simulation, and V° is the standard molar volume of the binding substrate.

Binding free energies from simulation

When calculating binding free energies from simulations, it is common to split up the overall binding process into two sub-processes, where one sub-process corresponds to the process of transferring the ligand from gas phase to the solvent, giving the solvation free energy, $\Delta G^{\circ}_{\mathrm{solv}}$, and the other sub-process corresponds to transferring the ligand from gas phase to the binding site of the receptor, forming a complex, giving a free energy which we denote as $\Delta G^{\circ}_{\mathrm{complex}}$ (Figure S3). Depending on how one constructs the thermodynamic cycle in order to obtain the binding free energy, these sub-processes can be constructed in different ways. In the double-annihilation method,[S6,S7] the sub-processes are constructed in reverse, so that the ligand is annihilated from the solvent in the first sub-process, and from the binding site in the second sub-process. Here, we construct the sub-processes in the forward direction, referred to as double creation of the ligand.[S8]

$$\begin{array}{rl}
\mathrm{B(g)} \rightarrow \mathrm{B(aq)} & \Delta G^{\circ}_{\mathrm{solv}} \\
\mathrm{A(aq)} + \mathrm{B(g)} \rightarrow \mathrm{AB(aq)} & \Delta G^{\circ}_{\mathrm{complex}} \\
\hline
\mathrm{A(aq)} + \mathrm{B(aq)} \rightarrow \mathrm{AB(aq)} & \Delta G^{\circ}_{\mathrm{bind}} = \Delta G^{\circ}_{\mathrm{complex}} - \Delta G^{\circ}_{\mathrm{solv}}
\end{array}$$

Figure S3: Scheme showing how the binding free energy is achieved in a simulation.

The double creation of the ligand is achieved by performing two molecular dynamics simulations in which the interactions of the ligand with the environment are gradually turned on, in the bulk and in the binding site, respectively.

Calculation of the standard binding free energy of transferring the ligand from the gas phase to the bulk solvent, $G^{\circ}_{\mathrm{solv}}$, is straightforward since it does not depend on the choice of standard concentration.[S9] Thus, the result one gets from a molecular dynamics simulation corresponds to the standard free energy of solvation, and no analytical corrections are needed. The reason is that the solvent is homogeneous, and as long as the interactions between the ligand and the solvent are captured, it should in principle not matter how large we make the

simulated system.

For the standard free energy of transferring the ligand from the gas phase to the binding site, $G^{\circ}_{\text{complex}}$, the scenario is different. The binding of a ligand to a receptor is associated with an entropic penalty, yielding a positive contribution to the binding free energy, which depends on the concentration of the ligand. One could capture this entropic penalty by letting the ligand sample the entire box numerous times and then count the number of times it binds to the receptor, and thereby obtaining the binding free energy, G°_{bind}. However, this is very inefficient, especially for systems with strong binding affinity in which configurations where the ligand is free are rarely sampled, and is thus typically not a feasible method. In addition, the ligand concentration we prepared the system with would have to equal the chosen standard concentration in order for this free energy measure to be a standard free energy.

This sampling issue is often circumvented by restraining the ligand in the binding site using a harmonic potential while turning on its interactions, yielding $\Delta G^{\text{site}}_{\text{complex}}$. Both translational and orientational restraints can be imposed, but here we are only interested in translational restraints (since the potassium ion has no orientational freedom). To account for the entropic penalty of restraining the ligand in the binding site one introduces a correction relating the accessible volume in the harmonic potential to the accessible volume in the simulation box. In this way, the free energy of transferring the ligand from the gas phase (now with a volume equal to that of the simulated system, V^{sim}) to the binding site, $G^{\text{sim}}_{\text{complex}}$, can be found as

$$\Delta G^{\text{sim}}_{\text{complex}} = \Delta G^{\text{site}}_{\text{complex}} + RT \ln \frac{V^{\text{sim}}}{V^{\text{site}}} \tag{S11}$$

Here, V^{site} is the volume of the binding site, which can be calculated by integrating the

Boltzmann factor of the restraining harmonic potential applied according to[S9]

$$V^{\text{site}} = \int_V \exp\left[-\frac{k(r_B - r_0)^2}{2RT}\right] d\mathbf{r}_B = \left(\frac{2\pi RT}{k}\right)^{3/2} \tag{S12}$$

where k is the force constant chosen for the harmonic potential, in units of J/mol/Å^2, and $r_B - r_0 = \delta r$ is the distance from the minimum in the harmonic potential (located at r_0). The force constant is chosen based on the mean positional fluctuation of the ligand in the binding site when it is unrestrained, $\langle \delta r^2 \rangle$,[S9]

$$k = \frac{3RT}{\langle \delta r^2 \rangle} \tag{S13}$$

The simulated binding free energy, corresponding to transferring the ligand from the bulk solvent to the binding site in the volume V^{sim} that defines the simulated system, can thus be written as

$$\begin{aligned} \Delta G^{\text{sim}}_{\text{bind}} &= \Delta G^{\text{sim}}_{\text{complex}} - \Delta G^{\circ}_{\text{solv}} \\ &= \Delta G^{\text{site}}_{\text{complex}} - \Delta G^{\circ}_{\text{solv}} + RT \ln \frac{V^{\text{sim}}}{V^{\text{site}}} \end{aligned} \tag{S14}$$

Finally, to obtain the standard binding free energy from the simulated free energies, corresponding to the standard molar volume V^0, the expression for $\Delta G^{\text{sim}}_{\text{bind}}$ in Equation S14 is inserted into Equation S10

$$\begin{aligned} \Delta G^{\circ}_{\text{bind}} &= \Delta G^{\text{site}}_{\text{complex}} - \Delta G^{\circ}_{\text{solv}} \\ &\quad + RT\left(\ln \frac{V^{\text{sim}}}{V^{\text{site}}} + \ln \frac{V^{\circ}}{V^{\text{sim}}}\right) \\ &= \Delta G^{\text{site}}_{\text{complex}} + \Delta G^{\circ}_{\text{solv}} + RT \ln \frac{V^{\circ}}{V^{\text{site}}} \end{aligned} \tag{S15}$$

where $\Delta G^{\text{site}}_{\text{complex}} + RT \ln \frac{V^{\circ}}{V^{\text{site}}} = \Delta G^{\circ}_{\text{complex}}$.

Multiple binding sites

For a receptor containing multiple identical and independent binding sites, the macroscopic binding constant K_i, corresponding to the binding of the i'th ligand to the receptor, can be written in terms of the intrinsic, or microscopic, binding constant, K_{intr}, according to[S10,S11]

$$K_i = \underbrace{\left(\frac{n-i+1}{i}\right)}_{=\ s_i} K_{\text{intr}} \tag{S16}$$

The factor in the parenthesis, s_i, is a statistical factor, where n is the total number of binding sites in the receptor, and i denotes the number in the series of binding events. The numerator in s_i is corresponding to the number of binding sites available for the i'th ligand. The denominator corresponds to the number of ligands that are bound when the i'th ligand has bound. For the system studied herein, the receptor (BCETB) possesses two binding sites ($n = 2$). Hence, there are two statistical factors, s_1 and s_2 , corresponding to the binding of the first and second potassium ion, respectively. The statistical factor reflects the ratio of

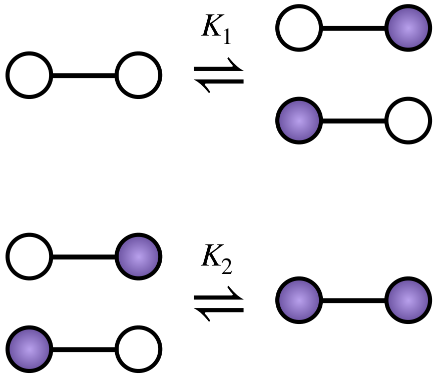

Figure S4: Simplified scheme showing the degree of degeneracy for states with zero, one, and two potassium ions bound to a receptor possessing two binding sites.

the degrees of degeneracy of the states before and after the binding occurs. As seen in Figure S4, there are two ways in which the state with one potassium ion bound can be achieved whereas the other states (zero and two potassium ions bound) are non-degenerate, yielding $s_1 = 2$ and $s_2 = 1/2$ for the first and second binding event, respectively.

In order to apply the double creation method to the problem at hand, including two binding sites, this effect has to be included. Since there are two binding events, the scheme as given in Figure S3 has to be extended to include three separate simulations as seen in Figure S5, where we have included chloride as the counterion. The first one predicts the free

$$\begin{array}{rlr} \mathrm{K^+(g) + Cl^-(g)} & \longrightarrow \mathrm{K^+(aq) + Cl^-(aq)} & \Delta G^\circ_{\mathrm{solv}} \\ \mathrm{BCETB\,(aq) + K^+(g) + Cl^-(g)} & \longrightarrow \mathrm{BCETB \bullet K^+(aq) + Cl^-(aq)} & \Delta G^\circ_{\mathrm{complex},1} \\ \mathrm{BCETB \bullet K^+(aq) + Cl^-(aq) + K^+(g) + Cl^-(g)} & \longrightarrow \mathrm{BCETB \bullet 2\,K^+(aq) + 2Cl^-(aq)} & \Delta G^\circ_{\mathrm{complex},2} \\ \hline \mathrm{BCETB\,(aq) + K^+(aq) + Cl^-(aq)} & \longrightarrow \mathrm{BCETB \bullet K^+(aq) + Cl^-(aq)} & \Delta G^\circ_{\mathrm{bind},1} \\ \mathrm{BCETB \bullet K^+(aq) + K^+(aq) + 2Cl^-(aq)} & \longrightarrow \mathrm{BCETB \bullet 2\,K^+(aq) + 2Cl^-(aq)} & \Delta G^\circ_{\mathrm{bind},2} \end{array}$$

Figure S5: Scheme showing how the binding free energy is achieved for the consecutive binding of two potassium ions to BCETB in a simulation, where $\Delta G^\circ_{\mathrm{bind},1} = \Delta G^\circ_{\mathrm{complex},1} - \Delta G^\circ_{\mathrm{solv}}$ and $\Delta G^\circ_{\mathrm{bind},2} = \Delta G^\circ_{\mathrm{complex},2} - \Delta G^\circ_{\mathrm{solv}}$.

energy of transferring a potassium chloride ion pair from the gas phase to the bulk solvent by gradually turning on the interactions of the ion pair with the pure solvent, giving $\Delta G^0_{\mathrm{solv}}$. As discussed in the previous section, this prediction is not depending on the standard state, and is thus straightforward to calculate. In the second simulation, the same procedure is followed, but now we include BCETB and have the potassium ion restrained in one of the binding sites while turning on its interactions. In the third simulation, the only difference is that one potassium ion is already bound in one of the binding sites while turning on the interactions of a second potassium ion in the other binding site (see Figure S6). The latter two simulations yield $\Delta G^{\mathrm{site}}_{\mathrm{complex},1}$ and $\Delta G^{\mathrm{site}}_{\mathrm{complex},2}$, respectively, because we have not yet corrected for the standard state. In addition, when simulating $\Delta G^{\mathrm{site}}_{\mathrm{complex},1}$, there is no possibility for the first binding potassium ion to enter the second binding site while it is being created, nor any possibility for it to leave its binding site when the second potassium ion is created in the second binding site during the simulation of $\Delta G^{\mathrm{site}}_{\mathrm{complex},2}$. Thus, the entropic effect stabilizing the state with one potassium ion bound and destabilizing the state with two potassium ions bound, as reflected in the statistical factors, is not captured, and

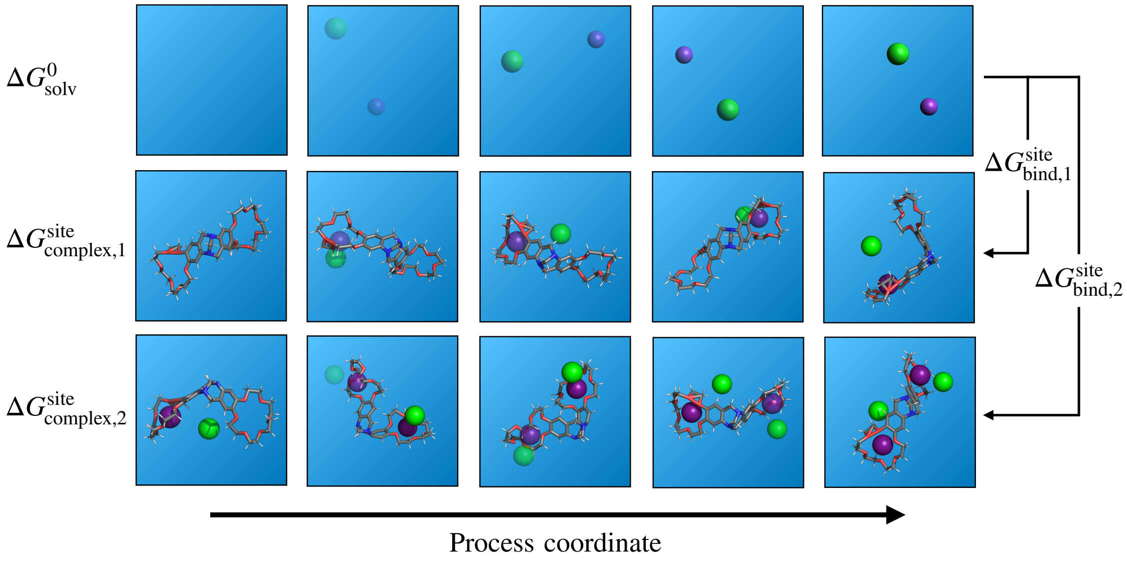

Figure S6: Scheme showing how the binding free energies for the two binding events are obtained using the double creation method. Each window represents a separate simulation, and by subtracting the solvation free energy, $\Delta G^{\circ}_{\text{solv}}$, from the complexation free energies, $\Delta G^{\text{site}}_{\text{complex,1}}$ and $\Delta G^{\text{site}}_{\text{complex,2}}$, the binding free energies, $\Delta G^{\text{site}}_{\text{bind,1}}$ and $\Delta G^{\text{site}}_{\text{bind,2}}$, are obtained.

we have to include the statistical factors as correction factors. Together with the correction relating the accessible volume in the binding site to the standard molar volume, this enables us to write the standard binding free energies for the two consecutive binding events as

$$\Delta G^{\circ}_{\text{bind,1}} = \Delta G^{\text{site}}_{\text{complex,1}} - \Delta G^{\circ}_{\text{solv}} + RT \ln \frac{V^{\circ}}{V^{\text{site}}} + RT \ln \frac{1}{2}, \tag{S17}$$

and

$$\Delta G^{\circ}_{\text{bind,2}} = \Delta G^{\text{site}}_{\text{complex,2}} - \Delta G^{\circ}_{\text{solv}} + RT \ln \frac{V^{\circ}}{V^{\text{site}}} + RT \ln 2. \tag{S18}$$

From these equations, we see that the corrections apply to $\Delta G^{\text{site}}_{\text{complex,1}}$ and $\Delta G^{\text{site}}_{\text{complex,2}}$, and we have

$$\Delta G^{\circ}_{\text{complex,1}} = \Delta G^{\text{site}}_{\text{complex,1}} + RT \ln \frac{V^{\circ}}{2V^{\text{site}}} \tag{S19}$$

and

$$\Delta G^{\circ}_{\text{complex,2}} = \Delta G^{\text{site}}_{\text{complex,2}} + RT \ln \frac{2V^{\circ}}{V^{\text{site}}} \tag{S20}$$

Finally, we obtain the difference between the standard binding free energies as

$$\begin{aligned}\Delta\Delta G^{\circ}_{\text{bind}} &= \Delta G^{\circ}_{\text{bind},2} - \Delta G^{\circ}_{\text{bind},1} = \Delta G^{\circ}_{\text{complex},2} - \Delta G^{\circ}_{\text{complex},1} \\ &= \Delta G^{\text{site}}_{\text{complex},2} - \Delta G^{\text{site}}_{\text{complex},1} + RT \ln 4\end{aligned} \tag{S21}$$

Computational details

In all simulations, we used geometric mixing rules for the pairwise LJ interactions. The atom names assigned for BCETB are shown in Figure S7. The OPLS-AA atom type assignment with corresponding LJ parameters (σ and ϵ) and modified charges are presented in Table S2 and Table S4. The net charges on BCETB and the ions for the different bound states and the different force fields are presented in Table S3, where the force field using polarized DFT charges (DFT,pol.) exhibits non-zero net charges on the complexes with one and two potassium ions bound, and charges less than unity for the bound potassium ions.

For the thiocyanate ion, we assigned LJ parameters and partial charges from a previously developed force field using Lorentz-Berthelot mixing rules for the pairwise LJ interactions.[S12] Since we used geometric mixing rules herein, we defined explicitly the pairwise LJ interactions for thiocyanate-water, thiocyanate-potassium, and thiocyanate-thiocyanate, in order to replicate the pairwise interactions from the original force field. The explicit interactions are listed in the supporting information provided electronically.

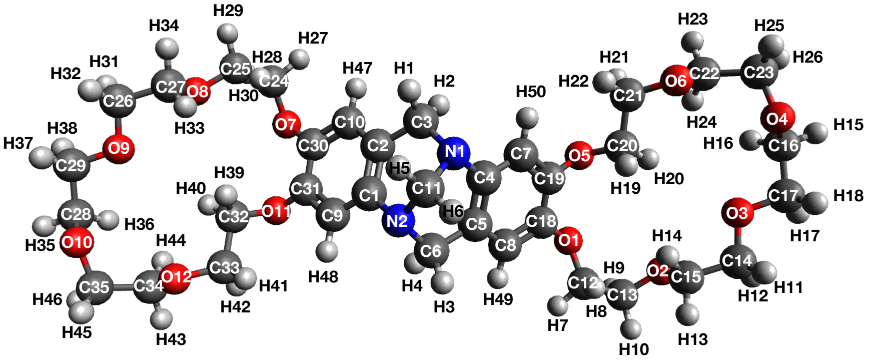

Figure S7: Atom names in BCETB.

Table S2: Atom type assignment and partial charges on BCETB for the different force fields (the partial charges on the polarized BCETB and K^+ can be found in the supporting information provided electronically).

atom	atom name(s)	type[a]	σ (Å)	ϵ (kJ/mol)	q_{OPLS} (e)	q_{DFT} (e)
C	C1, C4	opls_{918}	3.55	0.293	+0.2164646	+0.117318
	C2, C5	opls_{145}	3.55	0.293	−0.1085354	+0.212610
	C3, C6	opls_{908}	3.50	0.276	+0.0964646	−0.090455
	C7, C9	opls_{145}	3.55	0.293	−0.1085354	−0.126325
	C8, C10	opls_{145}	3.55	0.293	−0.1085354	−0.532066
	C11	opls_{908}	3.55	0.293	+0.0964646	−0.001379
	C12, C24	opls_{182}	3.50	0.276	+0.1464646	+0.036332
	C13, C25	opls_{182}	3.50	0.276	+0.1464646	+0.045991
	C14, C26	opls_{182}	3.50	0.276	+0.1464646	+0.033130
	C15, C27	opls_{182}	3.50	0.276	+0.1464646	+0.200612
	C16, C28	opls_{182}	3.50	0.276	+0.1464646	+0.118034
	C17, C29	opls_{182}	3.50	0.276	+0.1464646	+0.133605
	C18, C30	opls_{166}	3.55	0.293	+0.1564646	+0.461969
	C19, C31	opls_{166}	3.55	0.293	+0.1564646	+0.000398
	C20, C32	opls_{182}	3.50	0.276	+0.1464646	−0.025759
	C21, C33	opls_{182}	3.50	0.276	+0.1464646	+0.407006
	C22, C34	opls_{182}	3.50	0.276	+0.1464646	+0.127831
	C23, C35	opls_{182}	3.50	0.276	+0.1464646	+0.095335
N	N1, N2	opls_{902}	3.30	0.711	−0.6235354	−0.522747
O	O1, O7	opls_{180}	2.90	0.586	−0.3935354	−0.358393
	O2, O8	opls_{180}	2.90	0.586	−0.3935354	−0.494341
	O3, O9	opls_{180}	2.90	0.586	−0.3935354	−0.441770
	O4, O10	opls_{180}	2.90	0.586	−0.3935354	−0.491401
	O5, O11	opls_{180}	2.90	0.586	−0.3935354	−0.445769
	O6, O12	opls_{180}	2.90	0.586	−0.3935354	−0.560358
H	H1–H4	opls_{911}	2.50	0.063	+0.0664646	+0.135664
	H5, H6	opls_{911}	2.50	0.063	+0.0664646	+0.178042
	H7, H8, H27, H28	opls_{185}	2.50	0.126	+0.0364646	+0.113087
	H9, H10, H29, H30	opls_{185}	2.50	0.126	+0.0364646	+0.119377
	H11, H12, H31, H32	opls_{185}	2.50	0.126	+0.0364646	+0.089134
	H13, H14, H33, H34	opls_{185}	2.50	0.126	+0.0364646	+0.095819
	H15, H16, H35, H36	opls_{185}	2.50	0.126	+0.0364646	+0.076377
	H17, H18, H37, H38	opls_{185}	2.50	0.126	+0.0364646	+0.051360
	H19, H20, H39, H40	opls_{185}	2.50	0.126	+0.0364646	+0.064876
	H21, H22, H41, H42	opls_{185}	2.50	0.126	+0.0364646	+0.051360
	H23, H24, H43, H44	opls_{185}	2.50	0.126	+0.0364646	+0.105533
	H25, H26, H45, H46	opls_{185}	2.50	0.126	+0.0364646	−0.002658
	H47, H49	opls_{146}	2.42	0.126	+0.1214646	+0.052723
	H48, H50	opls_{146}	2.42	0.126	+0.1214646	+0.070783

[a] The LJ parameters were inherited from the assigned OPLS-AA atom types, but the partial charges were either shifted in order to make BCETB electroneutral (q_{OPLS}, used in the OPLS-AA force field), or determined from DFT calculations (q_{DFT}, used in the DFT force field).

Table S3: Net charges on BCETB and the ions during the coupling process of binding the first and the second K^+ for the different force fields.

compound	free BCETB	one K^+		two K^+	
	$q_{OPLS/DFT/DFT,pol.}$ (e)	$q_{OPLS/DFT}$ (e)	$q_{DFT,pol.}$ (e)	$q_{OPLS/DFT}$ (e)	$q_{DFT,pol.}$ (e)
BCETB	0.0000	0.0000	+0.1510	0.0000	+0.4889
first K^+	-	+1.0000	+0.8490	+1.0000	+0.7556
first $Cl^-/I^-/SCN^-$	-	−1.0000	−1.0000	−1.0000	−1.0000
second K^+	-	-	-	+1.0000	+0.7556
second $Cl^-/I^-/SCN^-$	-	-	-	−1.0000	−1.0000

Table S4: Atom type assignment and partial charges on ions and water for the different force fields.

compound	atom	type	σ (Å)	ϵ (kJ/mol)	q_{OPLS} (e)	q_{DFT} (e)
potassium	K^+	$opls_{408}$	4.93	0.001	+1.0000	+1.0000
chloride	Cl^-	$opls_{401}$	4.42	0.493	−1.0000	−1.0000
iodide	I^-	$opls_{403}$	5.40	0.293	−1.0000	−1.0000
SCN^- [S12]	S	-	3.83	1.523	−0.5730	−0.5730
	C	-	3.35	0.425	+0.4830	+0.4830
	N	-	3.70	0.310	−0.9100	−0.9100
SPC/E[S13]	O	$opls_{116}$	3.17	0.650	−0.8476	−0.8476
	H	$opls_{117}$	0.00	0.000	+0.4238	+0.4238

Table S5: Force constants and binding site volumes calculated based on the simulated mean squared positional fluctuation of the potassium ion in the binding site of BCETB using OPLS-AA charges (k_{OPL} and V^{site}_{OPL}), DFT charges (k_{DFT} and V^{site}_{DFT}), and polarized DFT charges ($k_{DFT,\ pol.}$ and $V^{site}_{DFT,\ pol.}$) at T = 298.15 K. For the elevated temperatures, the force constant determined at T = 298.15 K is scaled with the factor $T/298.15$, where T is the temperature of interest in units of K.

T (K)	k_{OPL} (kcal mol^{-1} Å^{-2})	V^{site}_{OPL} (Å^3)	k_{DFT} (kcal mol^{-1} Å^{-2})	V^{site}_{DFT} (Å^3)	$k_{DFT,pol.}$ (kcal mol^{-1} Å^{-2})	$V^{site}_{DFT,pol.}$ (Å^3)
298.15	8.60	9.00	9.56	7.68	2.39	61.5
302.15	8.72	9.00	9.69	7.68	2.42	61.5
306.15	8.84	9.00	9.82	7.68	2.45	61.5
310.15	8.95	9.00	9.95	7.68	2.49	61.5
314.15	9.07	9.00	10.07	7.68	2.52	61.5
318.15	9.18	9.00	10.20	7.68	2.55	61.5

The mean squared positional fluctuation of the potassium ion in the binding site, $\langle \delta r^2 \rangle$, was determined by running a simulation at $T = 298.15$ K in GROMACS with the harmonic potential active but with k set to zero, and then averaging the squared positional fluctuation of the bound potassium ion that the software outputs over time.[S9] In order to keep the volume of the binding site, V^{site}, constant for simulations performed at different temperatures, we further scaled the force constants used for the elevated temperatures with the factor $T/298.15$, where T is the temperature of interest in units of K. The force constants and the resulting binding site volumes determined using OPLS-AA charges, DFT-charges, and polarized DFT-charges are presented in Table S5.

Binding enthalpies

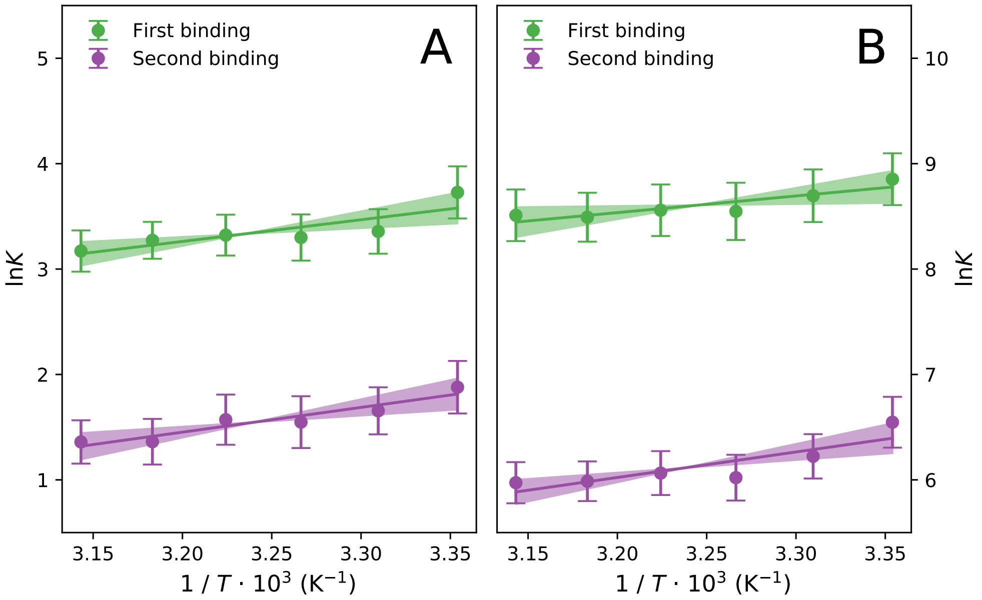

Figure S8: Binding free energies as a function of temperature obtained from simulations with KCl using OPLS-AA charges (A) and DFT charges (B). The symbols are simulated points, the lines are linear fits used to calculate the binding enthalpies, and the shaded areas are the standard errors of the slopes, yielding the error estimates for the binding enthalpies. Green and purple color show the results obtained for the first and second binding event, respectively.

For the binding enthalpies, the linearized van't Hoff Equation was applied on a range

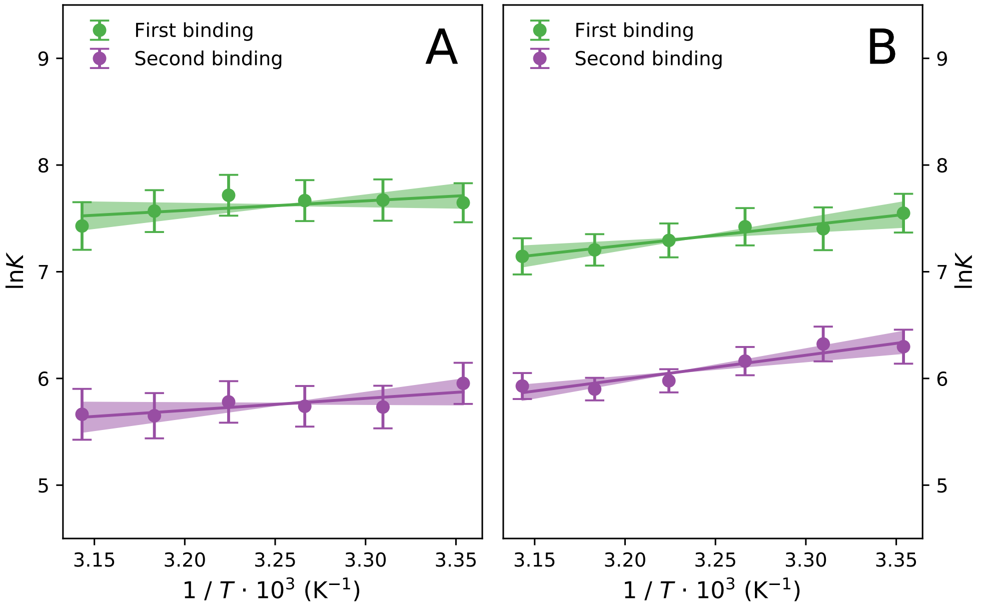

Figure S9: Binding free energies as a function of temperature obtained from REMD simulations with KI (A) and KSCN (B) using DFT charges. The symbols are simulated points, the lines are linear fits used to calculate the binding enthalpies, and the shaded areas are the standard errors of the slopes, yielding the error estimates for the binding enthalpies. Green and purple color show the results obtained for the first and second binding event, respectively.

of binding free energy estimates (T = [298.15, 302.15, 306.15, 310.15, 314.15, 318.15] K). The natural logarithm of the binding constant, $\ln K = -\Delta G^{\circ}_{\text{bind}}/(RT)$, was plotted against $1/T$, after which linear regression was performed on the set of data points. The slope is corresponding to $-\Delta H^{\circ}_{\text{bind}}/R$, assuming the enthalpy and entropy to be independent of temperature in the range used. To estimate errors on the binding enthalpies, the bootstrap method was employed.[S4] The free energy estimates were decomposed into 2.5 ns block estimates resulting in 12 blocks for $\Delta G^{\circ}_{\text{solv}}$ (30 ns in total), and 20 blocks for $\Delta G^{\circ}_{\text{complex}}$ (50 ns in total). New estimates of the binding free energies were obtained by random resampling of the block estimates, after which a new average was calculated. The resampling was performed with replacement, so that the same block estimate could occur several times, or be absent, in each new estimate. For each new set of free energy estimates, linear regression was repeated, yielding a new estimate of the binding enthalpy. The standard error of the enthalpy was finally obtained as the standard deviation of the enthalpies generated through resampling of the block estimates.

Conformational dynamics

The elucidate whether the conformational dynamics of BCETB is perturbed upon binding, we performed principal component analysis (PCA).[S14] As input, we used the pairwise distances between all heavy atoms in BCETB (all atoms except hydrogen). To map the most probable structures, we included the first two components (accounting for 81% of the variance), and counted the number of times each point was sampled in the PCA space. The result is a 2-dimensional histogram, and by subtracting from all points the least sampled point and taking the natural logarithm, we obtain a 2-dimensional free energy landscape, with points close to each other representing similar conformations. To estimate the weight of each conformation, we integrated the number of sampled points within ellipses enclosing each minimum and normalized with the total number of points. The weight of the remain-

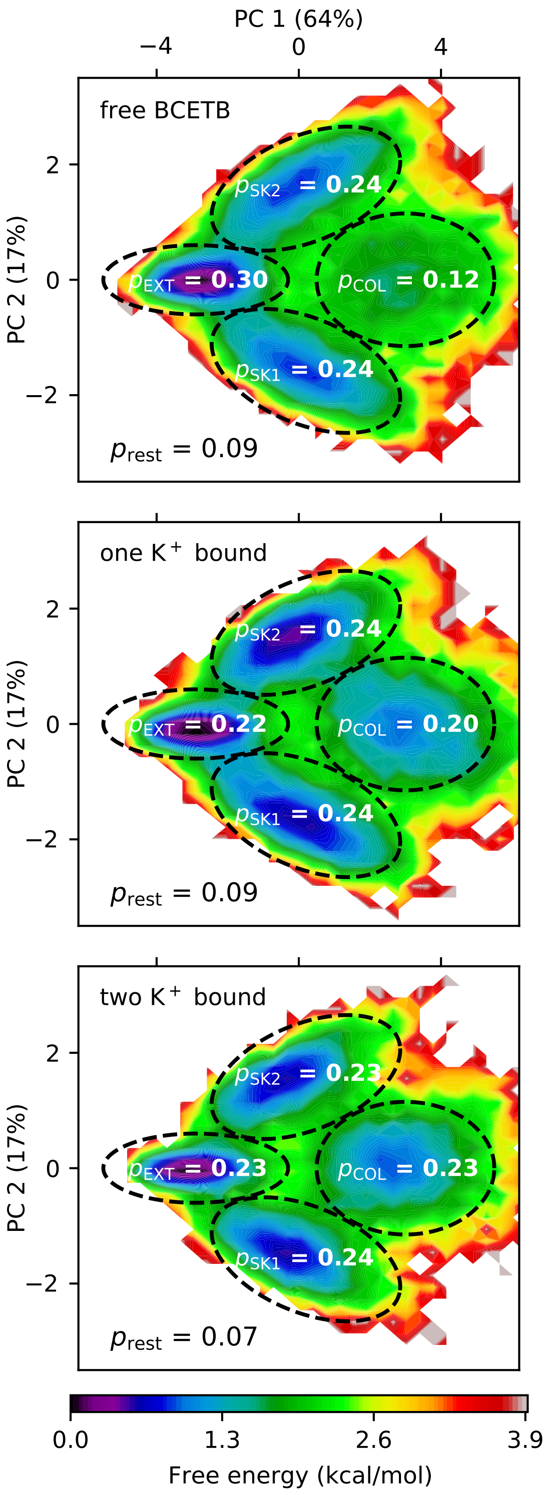

Figure S10: Principal component analysis conducted on all non-hydrogen pairwise atom distances in BCETB using DFT charges for the cases with no (top), one (middle), and two (bottom) K^+ bound in the system with potassium chloride.

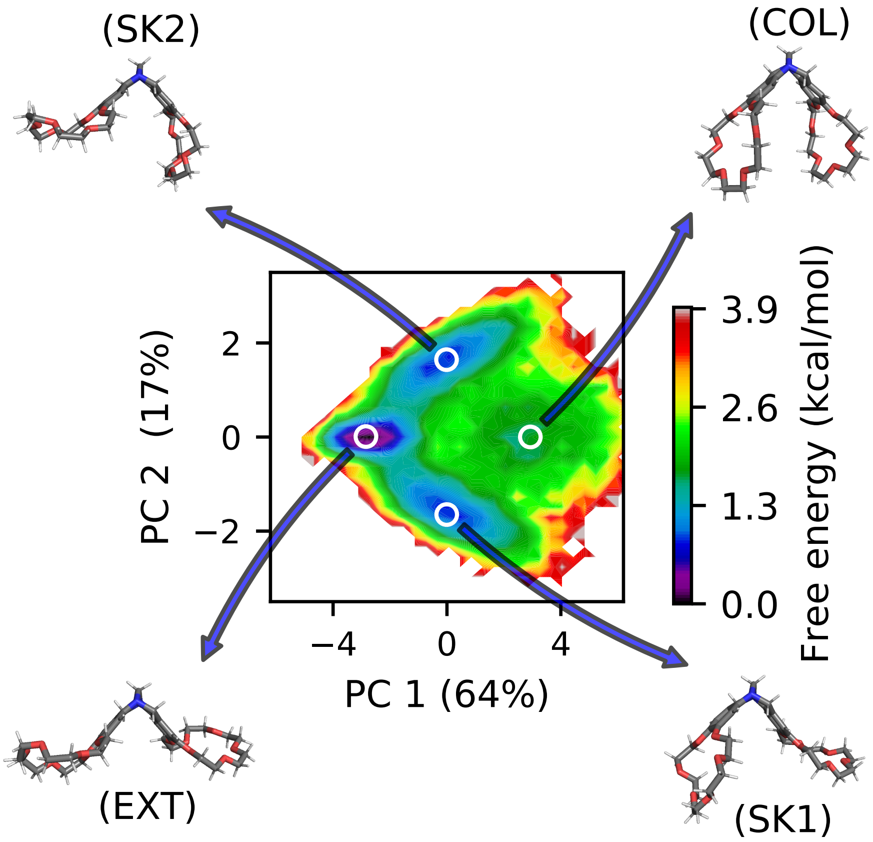

Figure S11: Principal component analysis conducted on all non-hydrogen pairwise atom distances in the free BCETB using DFT charges for the system with potassium chloride. The arrows indicate which conformation is corresponding to which minimum in PCA space.

der of the conformations (p_{rest} in Figure S10) was estimated by integrating the points not belonging to any of the ellipses.

Energies of the complexes and the conformations

The internal potential energies were calculated by averaging over all the configurations generated in the REMD simulations of the free BCETB, and the complexes with one and two potassium ions bound, respectively, but excluding the water and the counterions, using GROMACS 2019/4.[S15] In Table S6, the internal potential energies of the free BCETB (U_0) and the complexes with one and two potassium ions bound (U_1 and U_2) are presented. Table S7 shows the estimates of the probability weighted potential energies for all conformations (SK2, EXT, SK1, COL, and rest) and the different bound states. The differences in the probability weighted potential energies upon binding are shown in Table S8.

Table S6: Potential energies of the free BCETB (U_0), the complex with one K^+ bound (U_1), and the complex with two K^+ bound (U_2) using DFT charges. The standard errors are obtained using bootstrapping applied on 20 block estimates, each block corresponding to 5 ns of simulation time.

Force field	U_0 (kcal/mol)	U_1 (kcal/mol)	U_2 (kcal/mol)
DFT charges	175.30 ± 0.05	71.55 ± 0.05	−32.38 ± 0.05

Table S7: Potential energies of the conformations weighted with their respective probabilities (pU) using DFT charges.

Force field	Conformation	$(p_jU_j)_0$ (kcal/mol)	$(p_jU_j)_1$ (kcal/mol)	$(p_jU_j)_2$ (kcal/mol)
DFT charges	SK2	42.70 ± 3.81	17.37 ± 1.17	−7.55 ± 0.64
	EXT	53.37 ± 2.93	15.89 ± 1.16	−7.48 ± 0.49
	SK1	42.81 ± 4.73	17.39 ± 1.40	−7.70 ± 0.51
	COL	21.04 ± 1.61	14.29 ± 1.02	−7.52 ± 0.47
	rest	15.39 ± 0.76	6.61 ± 0.31	−2.12 ± 0.13

Table S8: Probability-weighted potential energy change upon binding of a first ($\Delta(pU)_{0-1}$) and a second ($\Delta(pU)_{1-2}$) potassium ion for the different conformations j (SK2, EXT, SK1, COL, or rest) using DFT charges. $\Delta\Delta(pU)$ equals the difference $\Delta(pU)_{1-2} - \Delta(pU)_{0-1}$.

Force field	Conformation	$\Delta(p_jU_j)_{0-1}$ (kcal/mol)	$\Delta(p_jU_j)_{1-2}$ (kcal/mol)	$\Delta\Delta(p_jU_j)$ (kcal/mol)
	SK2	-25.33 ± 3.94	-24.93 ± 1.33	0.40 ± 4.47
	EXT	-37.48 ± 3.15	-23.37 ± 1.26	14.11 ± 3.79
DFT charges	SK1	-25.42 ± 4.95	-25.09 ± 1.47	0.33 ± 5.56
	COL	-6.75 ± 1.89	-21.81 ± 1.12	-15.07 ± 2.62
	rest	-8.78 ± 0.82	-8.73 ± 0.33	0.05 ± 0.99

Solvent response

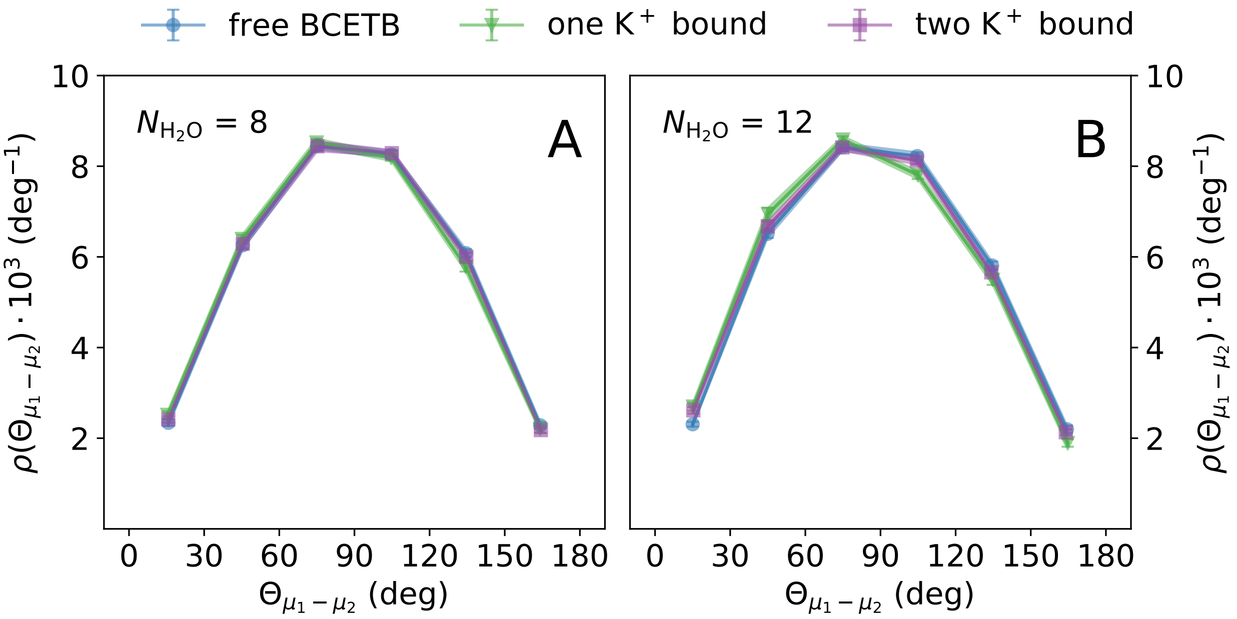

Figure S12: Probability densities of angles between the dipoles of two water clusters, one at each binding site in BCETB, from simulations with KCl using DFT charges. Plot A and B show the results for clusters containing 8 and 12 water molecules, respectively. The range of angles on the x-axis has been binned into sub-ranges, where each marker represents the probability density within angles $\pm$ 15° from the x-position of the marker (*e.g.* the leftmost markers show the probability densities of angles between 0° and 30°). The lines connecting the markers are merely to guide the eye, and the shaded areas show the interpolated errors between the probability densities for each sub-range.

According to continuum electrostatics theory, the solvent (water) is responsible for a negative enthalpy when bringing two positive charges closer in the solution bulk. Here, we

looked closer at the solvent response upon binding of the two potassium ions to BCETB to elucidate the impact of the receptor. By analyzing the alignment of the dipoles of two water clusters, one at each binding site, we gain molecular insight into how the binding of the first potassium ion affects the surrounding of the other binding site. In Figure S12, the distributions of angles between the dipoles of two water clusters, defined as the $N_{\mathrm{H_2O}}$ closest water molecules to each binding site, are plotted for $N_{\mathrm{H_2O}} = 8$, and $N_{\mathrm{H_2O}} = 12$.

Affinity of anions to BCETB

The distributions of anions and water molecules around BCETB were obtained by calculating and integrating over the radial distribution functions from the BCETB surface, using GROMACS 2019/4.[S15] The resulting numbers of ions or water molecules obtained as a function of the distance from the BCETB surface were normalized with the total number of ions/water molecules in the simulation box.

References

(S1) Freyer, M. W.; Lewis, E. A. *Biophysical Tools for Biologists, Volume One: In Vitro Techniques*; Elsevier, 2008; pp 79–113.

(S2) Izatt, R. M.; Terry, R. E.; Haymore, B. L.; Hansen, L. D.; Dalley, N. K.; Avondet, A. G.; Christensen, J. J. Calorimetric titration study of the interaction of several uni- and bivalent cations with 15-crown-5, 18-crown-6, and two isomers of dicyclohexo-18-crown-6 in aqueous solution at 25°C and $\mu = 0.1$. *Journal of the American Chemical Society* **1976**, *98*, 7620–7626.

(S3) Michaux, G.; Reisse, J. Solution thermodynamic studies. Part 6. Enthalpy-entropy compensation for the complexation reactions of some crown ethers with alkaline cations: a quantitative interpretation of the complexing properties of 18-crown-6. *Journal of the American Chemical Society* **1982**, *104*, 6895–6899.

(S4) Efron, B. Nonparametric estimates of standard error: The jackknife, the bootstrap and other methods. *Biometrika* **1981**, *68*, 589–599.

(S5) General, I. J. A Note on the Standard State's Binding Free Energy. *Journal of Chemical Theory and Computation* **2010**, *6*, 2520–2524.

(S6) Jorgensen, W. L.; Buckner, J. K.; Boudon, S.; Tirado-Rives, J. Efficient computation of absolute free energies of binding by computer simulations. Application to the methane dimer in water. *The Journal of Chemical Physics* **1988**, *89*, 3742–3746.

(S7) Gilson, M.; Given, J.; Bush, B.; McCammon, J. The statistical-thermodynamic basis for computation of binding affinities: a critical review. *Biophysical Journal* **1997**, *72*, 1047–1069.

(S8) Pohorille, A.; Jarzynski, C.; Chipot, C. Good Practices in Free-Energy Calculations. *The Journal of Physical Chemistry B* **2010**, *114*, 10235–10253.

(S9) Hamelberg, D.; McCammon, J. A. Standard Free Energy of Releasing a Localized Water Molecule from the Binding Pockets of Proteins: Double-Decoupling Method. *Journal of the American Chemical Society* **2004**, *126*, 7683–7689.

(S10) Hammes, G. G.; Wu, C. W. Kinetics of Allosteric Enzymes. *Annual Review of Biophysics and Bioengineering* **1974**, *3*, 1–33.

(S11) Perlmutter-Hayman, B. Cooperative binding to macromolecules. A formal approach. *Accounts of Chemical Research* **1986**, *19*, 90–96.

(S12) Tesei, G.; Aspelin, V.; Lund, M. Specific Cation Effects on SCN– in Bulk Solution and at the Air–Water Interface. *The Journal of Physical Chemistry B* **2018**, *122*, 5094–5105.

(S13) Berendsen, H. J. C.; Grigera, J. R.; Straatsma, T. P. The Missing Term in Effective Pair Potentials. *J. Phys. Chem.* **1987**, *91*, 6269–6271.

(S14) Pearson, K. LIII. On lines and planes of closest fit to systems of points in space. *The London, Edinburgh, and Dublin Philosophical Magazine and Journal of Science* **1901**, *2*, 559–572.

(S15) Spoel, D. V. D.; Lindahl, E.; Hess, B.; Groenhof, G.; Mark, A. E.; Berendsen, H. J. C. GROMACS: Fast, flexible, and free. *Journal of Computational Chemistry* **2005**, *26*, 1701–1718.
